# A rare allele of *TabZIP45-4B* enhances wheat adaptation to low nitrogen growth conditions

**DOI:** 10.1016/j.xplc.2026.101784

**Published:** 2026-02-26

**Authors:** Zhixiong Huang, Yazhou Wang, Hui Wang, Chuncai Shen, Guowei Chang, Wenjing Li, Mengyun Hu, Hui Li, Yijing Zhang, Wan Teng, Xueqiang Zhao, Longxi Zhou, Kang Zhang, Caixia Gao, Xue He, Yiping Tong

**Affiliations:** 1 State Key Laboratory of Seed Innovation, Institute of Genetics and Developmental Biology, Chinese Academy of Sciences, Beijing 100101, China; 2 Zhongweida PRAOVIDIA Biotechnology (Hangzhou) Co., Ltd., Hangzhou, Zhejiang 310030, China; 3 Hebei Laboratory of Crop Genetics and Breeding, Institute of Cereal and Oil Crops, Hebei Academy of Agriculture and Forestry Sciences, Shijiazhuang, China; 4 State Key Laboratory of Genetic Engineering, Collaborative Innovation Center of Genetics and Development, Department of Biochemistry, Institute of Plant Biology, School of Life Sciences, Fudan University, Shanghai 200438, China; 5 College of Advanced Agricultural Sciences, University of Chinese Academy of Sciences, Beijing 100049, China; 6 New Cornerstone Science Laboratory, Center for Genome Editing, Institute of Genetics and Developmental Biology, Chinese Academy of Sciences, Beijing 100101, China; 7 The Dryland Farming Institute, Hebei Academy of Agriculture and Forestry Sciences, Hengshui, China; 8 Institute of Chinese Materia Medica and Space Biotechnology, Jinhua Academy of Zhejiang Chinese Medical University, Jinhua 321032, China; 9 Yazhouwan National Laboratory, Sanya, Hainan 572024, China; 10 Genovo (Tianjin) Biotechnology Co., Tianjin, China

## Abstract

This study reports the identification of a rare allele of *TabZIP45-4B* (*TabZIP45-4B*^*pnd1*^) that confers tolerance to nitrogen starvation. Knockout of *TabZIP45-4B* increases spike number and grain yield per plant in wheat under nitrogen-starvation conditions by downregulating *TaDWARF4*. Introgression of *TabZIP45-4B*^*pnd1*^ into a modern commercial cultivar improves multiple agronomic traits with minimal nitrogen input.

Dear Editor,

Excessive nitrogen fertilizer use since the Green Revolution has imposed substantial environmental and economic burdens, with hidden costs exceeding US$1 trillion annually (FAO, 2023). Limiting nitrogen input markedly reduces tiller and spike numbers, resulting in significant yield penalties (Wu et al., 2020) and posing a critical challenge for sustainable agriculture. Although several nitrogen-responsive genes have been shown to regulate tiller and spike development—key indicators of nitrogen response—these genes likely represent only a fraction of a broader and more complex regulatory network (Wu et al., 2024). The mechanisms underlying the growth plasticity of spike number under nitrogen starvation therefore remain largely unresolved.

To uncover the genetic basis of adaptation to nitrogen limitation in wheat, we developed a backcross population (BC_5_F_5_–BC_5_F_10_) derived from two winter cultivars, Xiaoyan 54 (XY54) and Jing 411 (J411), which differ in nitrogen-use efficiency (NUE) (Xu et al., 2014). Segregating populations were generated from the near-isogenic lines (NILs) described above (Supplemental Figures 1A–1E). Genetic mapping identified a residual heterozygosity hotspot on chromosome 4B, designated *perception of nitrogen deficiency 1* (*pnd1*), whose genomic position coincides with intervals previously associated with nitrogen starvation (Xu et al., 2014). We next performed bulked segregant analysis by sequencing DNA and RNA from two extreme pools—one with high spike number and the other with low spike number—across three independent, NIL-derived populations (BC_4_F_5_–BC_4_F_10_ and BC_5_F_1_–BC_5_F_6_) segregating at the *pnd1* hotspot. Linkage mapping under nitrogen-limited conditions identified an approximately 390–410 Mb centromere-proximal region significantly associated with spike number per plant (Supplemental Figure 1F). To refine this interval, we developed a high-resolution single-nucleotide polymorphism (SNP) detection method capable of distinguishing trace allele frequencies in pooled DNA libraries, narrowing the candidate region to 393 kb (391,158,732–391,552,259 bp) (Figure 1A; Supplemental Figure 1G; Supplemental Table 1; see Supplemental Methods). Among more than 14,000 high-spike individuals, only a single SNP within this interval showed complete linkage with spike number and a significant difference in expression (Figure 1A; Supplemental Figures 1G–1I). Sequence analysis identified a G-to-T SNP in the coding sequence of the sole annotated transcription factor within this interval, *TraesCS4B02G178600*, resulting in a conserved glycine-to-valine (G→V) substitution within the Delay of Germination (DOG) domain (Figure 1A and 1B; Supplemental Figure 2D). Mutations in this domain have been reported to induce steric and conformational changes that may influence DNA-binding affinity and consequently transcriptional activity (Fantini et al., 2009).

**Figure 1:**
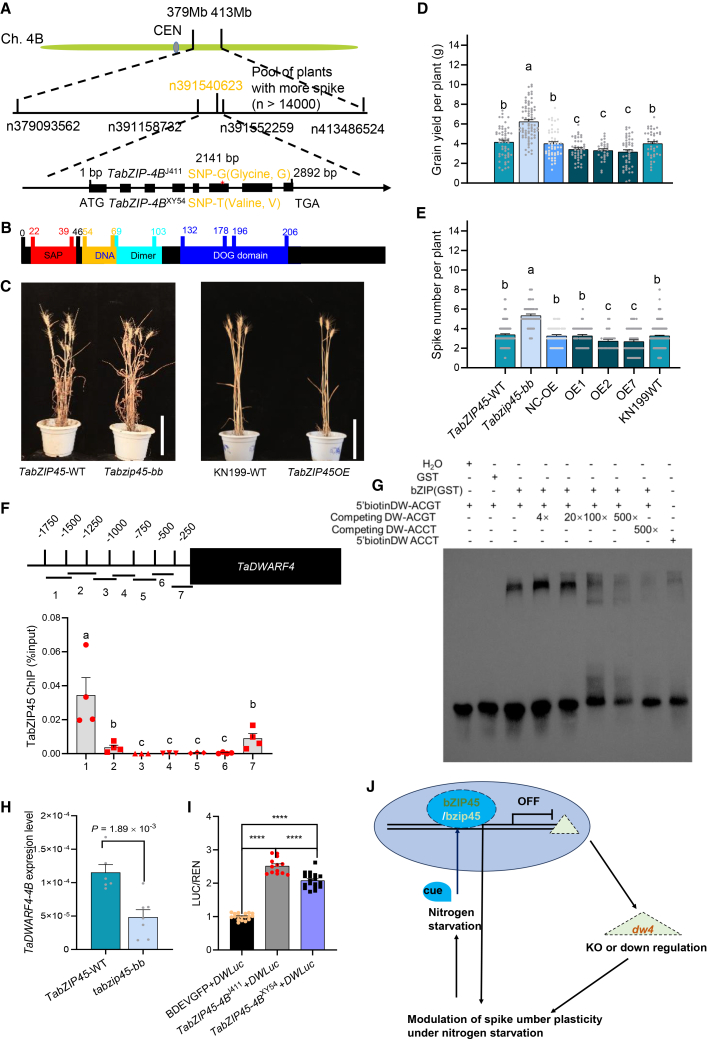
Molecular characterization of *TabZIP45-4B*/*Qpnd1* and its regulation of spike number under nitrogen starvation. **(A)** Positional mapping of the *pnd1* locus. The *pnd1* locus was originally mapped to a region spanning approximately 379–413 Mb **(**Supplemental Figure 1F**)**. Fine mapping further narrowed this region to a 393 kb interval containing only one single-nucleotide polymorphism (SNP) showing complete linkage disequilibrium within an annotated gene encoding a basic leucine zipper transcription factor **(**Supplemental Figure 1G**)**. The *pnd1* locus carries a G-to-T nucleotide transversion that results in a missense mutation from glycine (G) to valine (V). CEN, centromere; Ch., chromosome. “n” followed by numbers indicates the physical positions of SNPs. **(B)** Domain architecture and CRISPR–Cas9-generated mutation sites of TabZIP45. The protein contains a Ser-Ala-Pro (SAP) motif (red), DNA double-helix binding sites (brown), the bZIP45 dimer interface domain (cyan), and the Delay of Germination (DOG) domain (blue). Amino acid residue 178 differs among TabZIP45-4A, TabZIP45-4B, and TabZIP45-4D. Different haplotypes of TabZIP45-4B contain distinct amino acid residues at position 196. Genome-editing–induced insertions or deletions cause a frameshift beginning at residue 46 and produce a truncated protein. **(C–E)** Phenotypes **(C)** of the knockout mutant *Tabzip45-bb* (*TabZIP45*-*4B* [AA*bbDD*]), overexpression line (OE), transgenic recipient wild-type control (KN199WT), *TabZIP45-WT* (negative control of *Tabzip45*-bb, *TabZIP45*-*4B* [AA*BBDD*]), and NC-OE (the negative control of OE lines). Scale bars, 20 cm. Grain yield **(D)** and spike number **(E)** of all lines were measured under a nitrogen supply of 90 kg ha^−1^ (N90) in field trials. **(F)** TabZIP45 binds to the *TaDWARF4* promoter. ChIP–PCR indicates sequence-specific binding and validates the ChIP-seq results (*n* = 3–4). **(G)** Electrophoretic mobility shift assay showing TabZIP45 binding to the DW-ACGT site (competed by free probe) but not to the mutated DW-ACCT site. **(H)** Expression of *TaDWARF4-4B* in the shoots of *Tabzip45-bb* (mutant) and *TabZIP45-*WT (negative control of *Tabzip45-bb*) plants under 0.0 mM NH_4_NO_3_ (0N). **(I)***TabZIP45-4B* increases *DWARF4-4B promoter–luciferase* transcriptional activity in a haplotype-dependent manner. ∗∗∗∗*p* < 0.0001. **(J)** Proposed working model of *TabZIP45-4B* in regulating spike number plasticity under nitrogen starvation. Knockout (KO) of *TabZIP45-4B* and/or downregulation of *TaDWARF4* in *Tabzip45-bb* may contribute to increased spike number under nitrogen starvation. All data are presented as mean ± SEM. In **(D)–(F)**, different letters indicate statistically significant differences determined by one-way ANOVA followed by Fisher’s LSD test (*p* < 0.05). In **(H) and (I)**, statistical significance was determined using Student’s *t*-test (unpaired, two-tailed). All experiments represent biological replicates, and results are representative of at least three independent experiments.

This gene encodes a basic leucine zipper (bZIP) transcription factor, hereafter named TabZIP45-4B, which is homologous to mammalian CREB and the angiosperm Flowering Locus D (FD; AtbZIP14 in *Arabidopsis*) (Supplemental Figures 2 and 3). Global haplotype surveys revealed that the *TabZIP45-4B*^XY54^ (*pnd1*) variant is rare (Supplemental Figure 4). Multi-year, multi-site field trials showed that cultivars and landraces carrying the two major *TabZIP45-4B* haplotypes differ significantly in spike number per plant and exhibit a near-significant difference in thousand-kernel weight, whereas sterile spikelet number and grain number per main spike remain unaffected (Supplemental Figure 4B–4E). TabZIP45-4B is orthologous to TGACG-binding (TGA) transcription factors, including TGA2.2, TGA5, TGA6, and TGA8, which function in both abiotic and biotic stress responses (Supplemental Figures 1J, 2D, and 3). In hexaploid bread wheat (*Triticum aestivum* L.), three paralogs—*TabZIP45-4A*, *TabZIP45-4B*, and *TabZIP45-4D*—are located on chromosomes 4A, 4B, and 4D, respectively, with a distinctive amino acid substitution at position 178 unique to TabZIP45-4B (Figure 1B; Supplemental Figures 2D and 5A). To assess the function of *TabZIP45-4B* under nitrogen deficiency, we generated loss-of-function mutants (*Tabzip45-bb) using* CRISPR–Cas9 and overexpression lines (*TabZIP45-4B OE)* in the KN199WT background (recipient cultivar), which carries the *TabZIP45-4B*^XY54^ haplotype (Supplemental Figure 5B–5H; Supplemental Table 2). Under nitrogen-limited conditions (N30, 30 kg N ha^−1^), *Tabzip45-bb* mutants and *TabZIP45-4B* OE lines exhibited significant increases and decreases in grain yield, respectively. These changes were driven by variation in spike number and grain number per spike, whereas thousand-kernel weight remained unaffected (Figure 1C–1E; Supplemental Figures 6A and 6B). Together, these results indicate that *TabZIP45-4B* negatively regulates spike and grain numbers under nitrogen limitation.

To further investigate the role of *TabZIP45* in adaptation to nutrient limitation, we analyzed its expression under different nitrogen conditions. All three homoeologs (*4A*, *4B*, and *4D*) were upregulated under nitrogen limitation (0.2N vs. 2N) in both shoots and roots (Supplemental Figure 7A). Among them, *TabZIP45-4B* showed consistently higher basal expression (∼2–3-fold) across all nitrogen regimes. Tissue-specific profiling further revealed that *TabZIP45-4A* and *TabZIP45-4D* were induced only by nitrogen deprivation (0N) in roots, whereas *TabZIP45-4B* displayed a biphasic response to both nitrogen deficiency (0N) and excess (4N) (Supplemental Figure 7B). These results establish *TabZIP45-4B* as the predominant nitrogen-responsive homoeolog.

Subcellular localization analysis showed that *TabZIP45-4B* is exclusively localized to the nucleus (Supplemental Figure 8). To define its regulatory network under nitrogen limitation, we performed chromatin immunoprecipitation sequencing (ChIP-seq) and RNA sequencing (RNA-seq). Although bZIP proteins share a conserved basic leucine zipper domain (Figure 1B), they recognize diverse *cis*-elements, including the G-box (CACGTG), A-box (TACGTA), C-box (GACGTC), and the related T/G-box (CACGTT) (Fujii et al., 2000). ChIP-seq identified 835 *TabZIP45* binding sites within ±5 kb of transcription start sites, encompassing transcription factors, genes involved in lipid and brassinosteroid (BR) metabolism and transport, signaling components, and genes associated with nitrogen transport and assimilation (Supplemental Figure 9; Supplemental Table 3). Motif enrichment analysis revealed the canonical bZIP-binding BACGBNWN motif as the most overrepresented sequence, along with several additional enriched motifs (Supplemental Figure 9). Notably, prominent binding peaks were detected near cytochrome P450 genes, including *DWARF4*, which encodes a key enzyme in BR biosynthesis in cereals (Supplemental Figure 9B; Supplemental Tables 3 and 5) (Choudhary et al., 2012). RNA-seq analysis of the wild type and *Tabzip45-bb* seedlings under low-nitrogen (0.2N) conditions detected 88,617 expressed transcripts, among which 6535 transcripts from 6300 genes were significantly altered in the mutant (*p* < 0.05) (Supplemental Figure 10A; Supplemental Table 4). Gene ontology analysis highlighted cytochrome P450-mediated steroid-related metabolic processes as the most affected pathways (Supplemental Figure 10B).

We therefore hypothesized that TabZIP45-4B directly regulates *TaDWARF4*. Consistent with this hypothesis, ChIP-seq revealed binding of TabZIP45 to the *TaDWARF4-4B* promoter, and RNA-seq showed altered TaDWARF4 transcript levels in *Tabzip45-bb* compared with KN199WT (Supplemental Table 4). Electrophoretic mobility shift assays and *in vivo* ChIP–qPCR further confirmed the specific binding of TabZIP45-4B to the *TaDWARF4* promoter (Figure 1F and 1G). qPCR analysis demonstrated a significant reduction in *TaDWARF4-4B* expression in the mutant, whereas the expression of other homoeologs remained unchanged (Figure 1H; Supplemental Figures 9C and 9D). In addition, luciferase reporter assays indicated that TabZIP45-4B binds the BACGBNWN motif *in vivo* to activate transcription (Figure 1I). Together, these results establish TabZIP45-4B as a nitrogen-limitation–inducible transcriptional activator of *TaDWARF4*.

To assess the role of *TaDWARF4* in spike development under nitrogen limitation, we generated *TaDWARF4* triple mutants (*dw4abd*) in the hexaploid wheat cultivar KN199WT (Supplemental Figures 11A and 11B). Interestingly, *dw4abd* mutants displayed increased spike number and grain yield relative to KN199WT (Supplemental Figure 11C and 11D). These results are consistent with previous findings in rice, where *DWARF4* influences spike number and shoot architecture, thereby enhancing yield under nitrogen-limited conditions (Sakamoto et al., 2006).

BR metabolism is a central regulator of plant architecture and grain yield (Sakamoto et al., 2006). Compared with the wild type, *Tabzip45-bb mutants showed significant upregulation of* the cell-cycle marker *Mitotic Spindle Protein 1A*, accompanied by enrichment of genes associated with cell development programs (Supplemental Figure 12A–12D; Supplemental Table 6). Consistent with these transcriptional changes, mutation of *TabZIP45-4B* increased both the number and length of tiller buds, as observed in NIL^XY54^ compared with *TabZIP45*-WT (the negative control line of *Tabzip45-bb*, see below) and NIL^J411^, respectively (Supplemental Figures 12E–12H). In addition, BRs are known to enhance root foraging under low-nitrogen conditions, suggesting a potential link between *TabZIP45*-4B-mediated BR regulation and nitrogen-adaptive root growth (Jia et al., 2019). Consistent with this hypothesis, root growth was markedly enhanced in both *Tabzip45-bb* and NIL^XY54^, driven by increased root length and a higher number of root tips under nitrogen-limited conditions (Supplemental Figure 13). These findings further support the notion that TabZIP45-4B coordinates plant growth through regulation of *TaDWARF4*.

The rare allele *TabZIP45-4B*^*pnd1*^ in NIL^XY54^ conferred higher spike number, grain yield, and plant height compared with NIL^J411^ (Supplemental Figures 1A–1E). Motivated by this observation, we introgressed *TabZIP45-4B*^*pnd1*^ into the elite cultivar Jimai22 (JM22), which is cultivated on more than 23.3 million hectares. Leveraging our high-throughput haplotype-specific SNP discrimination technology, we accelerated the precise selection of *TabZIP45-4B*^*pnd1*^ introgression lines. As expected, JM22-*TabZIP45-4B*^*pnd1*^ lines outperformed the JM22 parent in spike number, grain yield, and grain number per spike without affecting plant height (Supplemental Figure 14).

Collectively, our results indicate that TabZIP45-4B is a previously uncharacterized nuclear regulator involved in adaptation to nitrogen deficiency. *TabZIP45-4B* transcriptionally regulates *TaDWARF4* and may help alleviate growth retardation under nitrogen-limited conditions. Through this regulation, TabZIP45-4B likely modulates BR pathways that are tightly coupled to shoot branching and tillering *in planta* (Hu et al., 2020), thereby quantitatively affecting spike number in wheat. Although the precise molecular intermediates linking TabZIP45-4B*-*dependent transcriptional changes to BR signaling dynamics remain to be elucidated, our genetic, transcriptional, and phenotypic analyses collectively delineate a coherent regulatory framework connecting nitrogen availability with growth plasticity in wheat.

In summary, this study demonstrates that knockout of *TabZIP45-4B* enhances spike number and grain yield per plant in wheat under nitrogen-starvation conditions by downregulating *TaDWARF4*. These findings indicate that transcriptional regulation of *TaDWARF4* by TabZIP45-4B plays an important role in modulating wheat growth in response to soil nitrogen availability. Notably, the rare allele *TabZIP45-4B*^*pnd1*^ identified in this study significantly improves the growth and yield performance of elite commercial cultivars under reduced nitrogen supply. Building on these findings, further investigation and targeted manipulation of the TabZIP45-4B*–TaDWARF4* regulatory module will deepen our understanding of the molecular networks governing plant adaptation to fluctuating soil nitrogen levels and provide valuable targets for developing cultivars with improved NUE, thereby contributing to more sustainable crop production worldwide.

## Funding

This work was supported by the National Key Research and Development Program of China (2022YFD1200204), the National Natural Science Foundation of China (U22A6009), and the Biological Breeding–National Science and Technology Major Project of China (2023ZD0402401).

## Acknowledgments

Z.H. is a co-founder of Hangzhou PRAOVIDIA Biotechnology Technology Industry Development Co., Ltd. (Hangzhou, China) and serves on its scientific advisory board. Z.H. has filed patent applications related to genomic marker selection and validation technologies, genome editing, and the identification of breeding targets using machine-learning and big-data mining approaches. Z.H. has also filed patent applications related to breeding and population-level seed quantification analytical methods associated with this study. In addition, Z.H. has filed patent applications for nucleotide discrimination technologies across multiple species, including applications in human disease samples, plants, animals, and microorganisms.

## Author contributions

Z.H. conceived the project, designed and performed experiments, analyzed data, and wrote and revised the manuscript with input from all other authors. C.S., W.T., Y.W., H.W., L.Z., G.C., X.Z., H.L., and M.H. assisted with data collection of wheat traits in field trials. C.G. and K.Z. developed plant materials. X.Z., C.S., and L.Z. assisted with genetic analyses. H.W., G.C., W.L., Y.Z., and X.H. assisted in manuscript revision. Y.T. initiated and designed the experiments, supervised the project, and acquired funding.

## Supplemental information

Supplemental Data 1. Supplemental Tables 1–8

Document S3. Article plus supplemental information
