# Supplementary material for: A rare allele of TabZIP45-4B enhances wheat adaptation to low nitrogen growth conditions
Source: Plant Commun. 2026 Feb 26;7(6):101784. doi: 10.1016/j.xplc.2026.101784 (PMC13261655; doi:10.1016/j.xplc.2026.101784)
Supplement: Document S1. Supplemental Figures 1–7, Supplemental Tables 1–8, and supplemental methods [file mmc1.pdf]

Plant Communications, Volume 7

Supplemental information

A rare allele of *TabZIP45-4B* enhances wheat adaptation to low nitrogen growth conditions

Zhixiong Huang, Yazhou Wang, Hui Wang, Chuncai Shen, Guowei Chang, Wenjing Li, Mengyun Hu, Hui Li, Yijing Zhang, Wan Teng, Xueqiang Zhao, Longxi Zhou, Kang Zhang, Caixia Gao, Xue He, and Yiping Tong

A rare alle of *TabZIP45-4B* enhances wheat adaptation to low nitrogen growth conditions

Zhixiong Huang[2,1*], Yazhou Wang[9], Hui Wang[8], Chuncai Shen[7], Guowei Chang[1], Wenjing Li[9], Mengyun Hu[3], Hui Li[3], Yijing Zhang[4], Wan Teng[1], Xueqiang Zhao[1], Longxi Zhou[1], Kang Zhang[10], Caixia Gao[1,5,6*], Xue He[1*]and Yiping Tong[1,5*].

[1] State Key Laboratory of Seed Innovation, Institute of Genetics and Developmental Biology, Chinese Academy of Sciences, Beijing 100101, China.

[2] Zhongweida PRAOVIDIA Biotechnology (Hangzhou) Co., Ltd., Hangzhou, Zhejiang 310030, China.

[3] Hebei Laboratory of Crop Genetics and Breeding, Institute of Cereal and Oil Crops, Hebei Academy of Agriculture and Forestry Sciences, Shijiazhuang, China

[4] State Key Laboratory of Genetic Engineering, Collaborative Innovation Center of Genetics and Development, Department of Biochemistry, Institute of Plant Biology, School of Life Sciences, Fudan University, Shanghai 200438, China

[5] College of Advanced Agricultural Sciences, University of Chinese Academy of Sciences, Beijing 100049, China.

[6] New Cornerstone Science Laboratory, Center for Genome Editing, Institute of Genetics and Developmental Biology, Chinese Academy of Sciences, Beijing 100101, China

[7] The dryland farming institute, Hebei academy of agriculture and forestry sciences, Hengshui, China

[8] Institute of Chinese Materia Medica and Space Biotechnology, Jinhua Academy of Zhejiang Chinese Medical University, Jinhua 321032, China.

[9] Yazhouwan National Laboratory, Sanya, Hainan 572024, China

[10] Genovo (Tianjin) biotechnology.co, Tianjin, China.

*Corresponding author. Email: hzxalan@yeah.net(Z.H.), yptong@genetics.ac.cn (Y.T.), hexue@genetics.ac.cn (X.H), cxgao@genetics.ac.cn (C.G)

Supplemental Figure 1. *TabZIP45-4B* alleles identification.

(A-E) Phenotypic comparison of near-isogenic lines (NILs) under nitrogen starvation conditions (30 kg N/ha). Grain yield **(A)**, spike number **(B),** height **(C),** grain number per spike **(D),** and thousand kernel weight (TKW) **(E)** of wheat under 30 kg N ha^{-1} (N30) in field trials at density of three independent paired near-isogenic lines (NIL-J411 and NIL-XY54). n ≥30.

(F) Gene position mapping of *TabZIP45-4B* in three independent inbreed near-isogenic populations from the NILs by bulked segregant analysis.

(G) Linkage disequilibrium (LD) analysis around locus of *pnd1*. The SNPs frequency (SNP ratio) in the narrowed physical positions of chromosome in **(F)**. The higher of significantly stably SNP ratio signal data points, the stronger LD of the SNP locus with the trait of productive spike number. Conversely, the random and/or wide-spread distributions of data points indicate weak LD between the SNP locus and the targeted trait. The SNP locus of 391540623 displayed complete LD (SNP frequency ratio = 1) which surrounding SNPs did not.

(H-I) Transcripts of *TabZIP45-4B* are changed by nitrogen supply levels. The gene expression of *TabZIP45-4B* in the root **(H)** (0.2 mM nitrogen, 0.2N) and shoot (**I**, n = 6-8) (2 mM nitrogen, 2N) of NILs (NIL-J411 and NIL-XY54). n = 6.

(**J**)*TabZIP45* is a *Arabidopsis* ortholog TGACG DNA-binding factors (TGA) *AtTGA2/6*, which are associated with a lot of genes/protein (in genetic /biochemistry) for plant growth and stresses response programs among chlorophyll content, meristem identity, cytoplasmic male sterility, diseases resistance, nitrate uptake, anthocyanin content, blast disease resistance, salt tolerance, oxidative stress, potassium concentration, aphid resistance, heat tolerance, stomatal resistance, root morphology trait, relative root length, yield trait and other miscellaneous traits.

All data are shown as mean ± s.e.m. In **A-C,** different letters indicate statistically significant differences by two-way ANOVA followed by (Fisher's LSD test, $P < 0.05$). In **D-E** and **H-I**, *P*-values number shows significant level by Student's *t* test (unpaired two-tailed). The experiments are all biological repeats, and results are representative of multiple independent experiments as needed (≥3).

Supplemental Figure 2. *TabZIP45* belongs to basic leucine zipper transcriptional factor superfamily.

(A) basic and zipper region (bZIP) superfamily.

(B) basic and zipper region conservation of cd1408bZIP subfamily.

(C) basic and zipper region conservation of whole bZIP subfamily.

(D) Arrows indicate the conservation of amino acid residues (aa) at 178 and 198 in both plants and mammals. The left above arrow indicates difference at 178 aa among TabZIP45-4A, -4B and -4D. The right arrow indicates the conserved 196 aa (Glycine, G) in DOG domain of TabZIP45-4B proteins. The left arrow points at dashed line red box at middle left side of panel indicates the position of methionine conservation between TabZIP45-4B and tumor related protein.

Supplemental Figure 3. TabZIP45 is an ortholog of AtTGA2 in evolutionary conserved analysis. The phylogeny tree was draw by neighbor-joining method of MEGA 6.0. The conserved amino residues between TabZIP45-4B and human CREB3.

Supplemental Figure 4. *TabZIP45-4B*^{XY54} (*pnd1*) is a rare allele of *TabZIP45-4B*.

(A) *TabZIP45-4B*^{XY54} (*Pnd1*) contributed to small proportion of global distribution of *TabZIP45-4B* alleles.

(B-E) Spike number per plant (B), thousand grain weight (C), sterile spikelet peri main spike (D) and grain number per main spike (E) of natural cultivars and landraces under 90 (N90), and 270 (N270) kg ha^{-1} in field trials. *P*-values number in B-E shows significant level by Student's *t* test (unpaired two-tailed). Data in B-E are shown as mean ± s.d. The experiments are all biological repeats, and results are representative of multiple independent experiments in Zhaoxian Hebei province, Dezhou Shandong province, Yuanyang Henan province at different years (2015-2019).

Supplemental Figure 5. Construction of *TabZIP45* genetic lines.

(A) TabZIP45-4B is different with TabZIP45-4A and TabZIP45-4D in amino acid residue site at 178. The sequences of proteins TabZIP45-4A, TabZIP45-4B and TabZIP45-4D were generated by DNAMAN (LynnonBiosoft, USA).

(B-C) Genome sequences and proteins sequences were disrupted by genome editing. Genome editing led to 8 bp insertion in *TabZIP45-4A,* one bp deletion in *TabZIP45-4B* and *TabZIP45-4D* respectively **(B).** Premature stop in TabZIP45-4A, 4B and 4D caused by genome editing produce 73 aa, 66 aa and 66 aa mutant proteins respectively **(C).**

(D-E) The genetic **(D)** and proteins background **(E)** of cultivar transgenic recipient cultivar KN199WT, homozygous *TabZIP45-4B* mutant of *Tabzip45-bb* (bb), homozygous *Tabzip45-aabbdd* (abd)mutant of *TabZIP45-4A4B4D*, positive DNA marker (P). antibody of TabZIP45: Anti-TabZIP45, antibody of heat shock protein 90: Anti-HSP.

(F-G) The genome sequences results of two independent *TabZIP45-4B* lines by CRISPR mutation (-1: deletion, +30: insertion).

(H) *TabZIP45* expression levels in transgenic recipient KN199WT wild type, negative control line of OE (NC-OE) is separated from OE transgenic heterozygotes without exogenous gene transformation after genotyping, which is strictly control line for OE relative to transgenic recipient KN199WT, and OE transgenic lines.

In **H** different letters indicate statistically significant differences by one-way ANOVA followed by (Fisher's LSD test, $P < 0.05$). All data are shown as mean ± s.e.m. The experiments are all biological repeats, and results are representative of multiple independent experiments as needed (≥3).

Supplemental Figure 6. *TabZIP45* exerts little effects on grain number per spike and thousand grain weight.

(A) The grain number per spike and **(B)** thousand grain weight of knockout line (*Tabzip45-bb*), overexpression line (OE), wildtype transgenic recipient control (KN199WT) and negative control (*TabZIP45*-WT control line of knockout and NC-OE negative line of OE) under limited nitrogen supply of 90 (N90) kg ha^{-1} in field trials.

All data are shown as mean ± s.e.m. The experiments are all biological repeats, and results are representative of multiple independent experiments as needed (≥3). In **A-B** different letters indicate statistically significant differences by one-way ANOVA followed by (Fisher's LSD test, $P < 0.05$). KN199WT is transgenic recipient cultivar. *TabZIP45*-WT [*TabZIP45-4B* (AA*BBDD*)] line is homogeneous offspring separated from heterozygotes of *TabZIP45-4B* (AA*BbDD*) knock out lines, which should be strictly control line for knock out line *Tabzip45-bb* [*TabZIP45-4B* (AA*bbDD*)] relative to KN199WT. NC-OE, Negative control line (of OE) is separated from OE transgenic heterozygotes without exogenous gene transformation after genotyping, which is strictly control line for OE relative to KN199WT.

Supplemental Figure 7. Nitrogen limitation induces gene expression of *TabZIP45*. **(A)** Transcripts of *TabZIP45* at 0.2N and 2N in shoot and root (n = 8) for 1 month. **(B)** Transcripts of *TabZIP45* at 0N, 1N (1 mM nitrogen), 2N and 4N (4 mM nitrogen) in root (n = 3-7) for 2 weeks.

In **A-B** different letters indicate statistically significant differences by one-way ANOVA followed by (Fisher's LSD test, $P < 0.05$). All data are shown as mean ± s.e.m. The experiments are all biological repeats, and results are representative of multiple independent experiments as needed (≥3).

Supplemental Figure 8. TabZIP45-4B is localized to nuclei of wheat protoplasts. Left column indicates signals from nuclei marker of 4',6-diamidino-2-phenylindole (DAPI) or chloroplast autofluorescence. Middle column indicates Green Fluorescent Protein (GFP). Right column indicates merge of pictures in previous two columns. TabZIP45-4B indicated TabZIP45-4B-GFP protein signal. GFP-EV indicated GFP empty vector. Scale bar, 5 µm.

Supplemental Figure 9. TabZIP45 downstream binding motifs and targets identification.

(A) The DNA binding motif sequence of *TabZIP45-4B* from chromatin immunoprecipitation sequencing-seq (ChIP-seq) enrichment.

(B) Presentative of *TabZIP45* regulation on its direct targets, *TaDWARF4*. ChIP peaks and input signals were identified with MACS2.

(C) The expression of *TaDWARF4-4A* in *Tabzip45-bb* (mutant) and *TabZIP45*-WT (negative control of *Tabzip45-bb* mutant) at 0.0 mM NH_4NO_3 (0N).

(D) The expression of *TaDWARF4-4D* in *Tabzip45-bb* (mutant) and *TabZIP45*-WT (negative control of *Tabzip45-bb*) at 0.0 mM NH_4NO_3 (0N).

In **C-D**, *P*-values number shows significant level by Student's *t* test (unpaired two-tailed). All data are shown as mean ± s.e.m. The experiments are all biological repeats, and results are representative of multiple independent experiments as needed (≥3).

Supplemental Figure 10. BRs-related-steroid metabolism programs are significantly enriched in the profiling of *TabZIP45* downstream gene expression. **(A)** The knock out of *TabZIP45-4B* changed genes expression fold change ≥ 10 significantly. The volcano plot showed genes with higher (red) or lower (Kalkavan et al.) genes expression in the root of *Tabzip45-bb* (versus KN199WT) after treatment with 0.1 mM NH_4NO_3 (0.2N) for two weeks

(B) Steroids biosynthesis programs were enriched in KEGG analysis.

Supplemental Figure 11. Mutation of *TaDWARF4* promotes plants growth under nitrogen limited conditions.

(A) Mutation sites of *TaDWARF4* triple mutants (*dw4abd*) by CRISPR-Cas9 genome editing.

(B-D) The phenotype of the *dw4abd* under nitrogen starved field conditions at nitrogen level of N0 (0 kg N/ha) with planting distance of 3 cm between two plants. The plant appearance **(B),** grain yield **(C)** and spike number **(D)** of *dw4abd* and KN199WT. In **C-D**, *P*-values number shows significant level by Student's *t* test (unpaired two-tailed). All data are shown as mean ± s.e.m. The experiments are all biological repeats, and results are representative of multiple independent experiments as needed (≥3). All data are shown as mean ± s.e.m. Scale bar, 20 cm.

Supplemental Figure 12. *TabZIP45-4B* regulates tiller initiation and elongation. **(A)** The relative expression level (TPM) of *Mitotic spindle protein 1A* in *TabZIP45*-WT and *Tabzip45-bb* under high N conditions (1.0 mM NH4NO3, HN) and/or (0.1 mM NH_4NO_3, LN) for two weeks and samples were collected.

(B-C) Genes set enrichment analysis (up regulated programs). **(B)** The mutation of *TabZIP45-4B* significantly up enriched gene expression program of development and growth. **(C)** The mutation of *TabZIP45-4B* significantly up enriched cell growth and developmental programs under nitrogen limited conditions.

(D) Ridge plot of genes expression programs of growth and development. GO

analysis was enriched and changed by the knockout of *TabZIP45-4B* after treatment with 0.1 mM NH_4NO_3 (0.2N) as indicated by dark green rectangle.

(E-H) Tiller buds number and tiller buds length of *TabZIP45*-WT and *Tabzip45-bb* **(E-F)**, NIL-*bZIP45-4B*^{J411} and NIL-*bZIP45-4B*^{XY54}**(G-H)**. Seedlings were grown under nitrogen limited (0.1 mM NH_4NO_3, LN) conditions. n = 8.

Data are mean ± S. E. *P* values in E-H were from paired Student's *t*-test. *TabZIP45*-WT, homozygous wild type offspring. *Tabzip45-bb*, homozygous mutant offspring. Isogenic lines NIL-*bZIP45-4B*^{J411} and NIL--*bZIP45-4B*^{XY54} are generated by introducing *TabZIP45-4B*^{J411} into XY54 (BC_5F_5). All data are shown as mean ± s.e.m. The experiments are all biological repeats, and results are representative of multiple independent experiments as needed (≥3).

Supplemental Figure 13. *TabZIP45-4B* modulates root growth plasticity to nitrogen perturbation.

(**A-D**) The knockout of *TabZIP45-4B* improved seedlings growth under limited nitrogen conditions. The root weight **(A)** and the ratio of root fresh weight / shoot fresh weight RFW/SFW **(B)** were increased in *Tabzip45-bb* (versus *TabZIP45*-WT) after nitrogen levels of 0.2 mM nitrogen (0.2N) for 2 months. The maximal root length **(C)** and shoot fresh weight **(D)** were not significantly changed.

(E-H) The rare allele of *TabZIP45-4B*^{XY54} significantly improved seedlings growth under limited nitrogen conditions. The root weight **(E)** and the ratio of RFW/SFW (**F**) were significantly increased in *TabZIP45-4B*^{XY54} (versus *TabZIP45-4B*^{J411}) after nitrogen levels of 0.2 mM nitrogen (0.2N) for 2 months. The maximal root length (**G**) and shoot fresh weight **(H)** were not significantly changed.

(I-K) The knockout of *TabZIP45-4B* changed root growth plasticity. The total root length **(I)**, total lateral root length **(J),** and tips number per seedling (**K**) of KN199WT and *Tabzip45-bb* under 2N and 0.2N. n = 12.

In **A-H**, *P*-values show significant level by Student's *t* test (unpaired two-tailed). In **I-K,** different letters indicate statistically significant differences by two-way ANOVA followed by (Fisher's LSD test, $P < 0.05$, n = 12). In **A-K**, data are shown as mean ± s.e.m. The experiments are all biological repeats, and results are representative of multiple independent experiments as needed (≥3).

Supplemental Figure 14. Introgression of *TabZIP-4B*^{pnd1} (*TabZIP45-4B*^{XY54}) improves elite modern cultivar Jimai22 (JM22) agronomic traits in Hebei province (Shijiazhuang) under field conditions.

(A) The plant appearance of JM22 and JM22-*TabZIP-4B*^{pnd1} under 0 kg N ha^{-1} (N0) at field population level. Scale bar = 20 cm.

(B) The spike number of JM22 and JM22-*TabZIP-4B*^{pnd1} (four independent lines) under nitrogen supply of 0 kg N ha^{-1} (N0) at field population level.

(C-F) *TabZIP-4B*^{pnd1} increased grain yield per plant **(C)** n ≥ 9, grain number per spike **(D)** n ≥ 9, without effects on plant height **(E)** n ⩾ 36 under 30 kg N ha^{-1} of line 6203.

All data are shown as mean ± s.e.m. **B-E**, *P*-values show significant level by Student's *t* test (unpaired two-tailed). The experiments are all biological repeats, and results are representative of multiple independent experiments as needed (≥3).

Methods

Pant material and growth condition

The winter wheat varieties Xiaoyan 54 (XY54), Jing 411 (J411), Kenong2011 (KN2011), Jimai22. BC_4F_{5-10} and BC_5F_{1-6} near-isogenic lines of J411//XY54, and BC_4F_{5-8} near-isogenic lines of KN2011// Jimai22were planted in the long-term low-nitrogen trial area (2015-2025 growing season) at the Beijing Changping Experimental Base of the Institute of Genetics and Developmental Biology, Chinese Academy of Sciences, and the long-term low-nitrogen and high nitrogen supply trial area (2015-2025 growing season) at the Zhao County Modern Agricultural Park Base in Shijiazhuang, Hebei, China. Transgenic recipient parental cultivar Kenong 199 (KN199WT) and each transgenic line: *TabZIP45* knockout lines *Tabzip45-bb*, *Tabzip45-aabbdd*, *TabZIP45*-AABBDD (*TabZIP45*-WT, negative control of knockout mutants), were grown at the Experimental Station of Cereal and Oil Crop Research Institute of Hebei Provincial Academy of Agricultural and Forestry Sciences, Gao Cheng District, Shijiazhuang, China. The gene editing lines were identified from T0 generation to obtain pure lines with each partial homozygous allele, or pure lines and negative control lines were isolated from heterozygous lines in T0 generation. During our screening for mutants with improved growth phenotypes under nitrogen-limiting conditions after field sowing, we obtained a heterozygous mutant line of *TabZIP45-4B*. Sequencing revealed a stop-gain mutation in the mutated *Tabzip45-Bb*. A total of 157 offspring from heterozygous mutant displayed a segregation ratio of 38 wild-type control plants (*TabZIP45*-WT): 84 heterozygous mutant plants (*Tabzip45-Bb*): 35 homozygous mutant plants (*Tabzip45-bb*) (approximately 1: 2: 1). The tobacco material used for transient expression cotransformation experiments was *Nicotiana benthamiana*.

Hydroponic culture condition

The common wheat culture was carried out as previously described with slight modifications (He et al., 2015). Seeds were germinated in 0.05% hydrogen peroxide overnight. The seeds were spread evenly to germinating in moist condition. Seven-day-old seedlings were transferred to plastic pots containing one-liter nutrient solution (0.2 mM KH_2PO_4, 1.5 mM KCl, 1.0 mM $MgSO_4.7H_2O$, 1.5 mM $CaCl_2$, 1.0 µM H_3BO_3, 0.05µM $(NH_4)_6Mo_7O_{24}.4H_2O$, 0.05µM $CuSO_4.5H_2O$, 1.0 µM $ZnSO_4.7H_2O$, 1.0 µM $MnSO_4.H_2O$, 0.1 mM FeEDTA(Na), 1.0 mM NH_4NO_3). The growth

condition was in in 16 hours day - 8 nigh hours cycle at 23°C. The different nitrogen supply levels were 0N (0.0 mM NH_4NO_3), 1N (0.5 mM NH_4NO_3), 2N (1.0 mM NH_4NO_3), 4N (2.0 mM NH_4NO_3). All the nutrition solution were refreshed every 4 days and pH was adjusted to 6.0.

DNA and RNA-seq and gene mapping by sequencing

To isolated genes that were responsible for coordination for growth and environmental response, we constructed a BC_5F_6 population by crossing between cultivar Jing411(a traditional Chinese winter wheat cultivar) and cultivar XiaoYan54 (XY54, a widely used cultivar in breeding) to generated NILs (NIL-XY54 and NIL-J411). The RNA and DNA from three paired-pools (one was with more tiller number and the other was less tiller number) were collected from offsprings of the NILs. There paired inbreed lines from $BC_4F_{5\text{-}10}$ and $BC_5F_{1\text{-}6}$ (Line27duo-line27shao, line28duo-line28shao, and line2020duo-line2020shao) seedlings at flowering stage were collected and RNA was isolated by GeneMarkbio Plant Total RNA Purification Kit (TR02). The cDNA from reverse transcription was sent to library construction. DNA was extracted by CTAB methods at same time. Hiseq-PE150 was used to sequence each library.

BSA/R methods were used to mapped physical location of loci (Huang et al., 2022; Soyk et al., 2017). The data from sequenced was filtered by Trimmomatic (Bolger et al., 2014). The resulting sequences were aligned to common wheat genome reference 2.0 by tophat2 (Kim et al., 2013) (--mate-std-dev 50 --min-coverage-intron 5 --min-segment-intron 5) and sorted, marked and indexed by samtools (Li et al., 2009). SNPs were called with samtools/bcftools pipeline(Li, 2011). The SNPs were filtered by samtools mpileup(Li *et al.*, 2009) (-Bugp -R -t DP,AD,ADF,ADR,SP,INFO/AD,INFO/ADF,INFO/ADR -I -q 50 -Q 14 -s) and bcftools and further filtered by SWEEP when necessary (Clevenger and Ozias-Akins, 2015). The SNP depth ratios in different paired pools were calculated according to methods as previously described (Soyk *et al.*, 2017)**.** In addition, we confirmed our results by software Triti-Map as previous described (Zhao et al., 2022).

The use of spike-number ratios under contrasting nitrogen regimes, as has proven effective in flooded rice systems where tiller counts are inherently stable. In wheat, however, field-scale heterogeneity in soil fertility and microenvironment makes it nearly impossible to distinguish genuine genetic effects from local edaphic variation when using ratio metrics; such variability also contributes to the historical difficulty in cloning spike-number QTLs in wheat. Because we observed larger variation in spike number under limited nitrogen conditions (An et al., 2006; Ma et al., 2023; Xu et al., 2014; Zhang et al., 2017)(Zhang, 2017 #659), we focused our mapping efforts exclusively on the nitrogen-limited phenotypes, selecting only the highest-performing individuals (“extremes”) for bulked segregant analysis. To avoid misleading, we would like to clarify that gene isolation under nitrogen deprivation conditions in this study is slightly different to previous reports on nitrogen responding gene isolation.

This extreme-pooling strategy leverages the expectation that, at a true causal locus, the favorable allele will approach fixation in the targeted traits (such as pool of individuals with high-spike number), while unlinked markers remain at background frequencies. By combining this approach with our high-resolution ultra-high throughput SNP discrimination technologies we developed based previous reports (Johnson et al., 2004; Martino et al., 2010; Taira et al., 2011; Zhang et al., 2020), we narrowed the candidate interval from 33 genes to a single nonsynonymous SNP in the exon of *TabZIP45-4B* in short time, consequently accelerating of our mapping and introgression.

For fine-mapping of genetic loci. SNP ratio between two haplotypes was calculated in population to narrow the genetic range. Briefly, kits from (P002) and primers were designed by Hangzhou Zhongweida PRAOVIDIA Biotechnology (Hangzhou) Technology Industry Development Co., Ltd, China [Nanxiang Village, Nanyang Sub-district, Xiaoshan District, Hangzhou City,

Zhejiang Province, 311227] were used to determine gene ratio. Allele specific primers (Supplemental Table 1) was used to determined Ct value in the DNA pool from individuals with more spike numbers. The SNP ratio in population (p) was determined as zp.

$$zp = 2e^{Ct(target-haplotype-in-p)-Ct(target-internal-in-p)}$$

Similarly, the SNP ratio in haplotype1 (1) of hybrid parents was determined as $z1$.

Similarly, the SNP ratio in haplotype2 (2) of hybrid parents was determined as $z2$.

Supplemental Table S7 │the robustness of high-resolution of trace DNA discrimination technology

	z1	z2	Theoretical range (z1/z2~z2/z1)	Detection Resolution	Max detection capacity per billion
Our methods	$1.37082E^{-05}$	$6.38169E^{-11}$	$4.66E^{-06}$~$2.15E^{+05}$	$1/(4.61e^{10})$	1 reaction
traditional methods	Unable to obtain	Unable to obtain	Unable to obtain	Unable to obtain	1 billion reactions

Plasmid construction and transformation

Wheat overexpression transgenic vector: *proTabZIP45*::*TabZIP45* was constructed on 163-JIT backbone (kindly donated by Caixia Gao, IGDB). The above constructed vectors were entrusted to our transformation platform to transform the wheat variety KN199WT using the gene gun transformation method as previously described (Shan et al., 2014). Independent transgenic lines were obtained and primers (one end on the vector sequence and the other end on the exogenous sequence) were set at the 5' and 3' ends of the respective transgene exogenous sequences, respectively. Positive plants were identified when the size of the sequence fragments amplified at both ends matched that of the plasmid positive control (verified by Sanger sequencing if necessary) and verified by sequencing. Expression was also identified in field or hydroponic conditions to confirm the success of the respective transgenic events.

Electrophoretic mobility shift assay (EMSA)

EMSA was carried out as previously described (Li et al., 2020). The full length of *TabZIP45-4B* was clone in to the *PGEX-4T-1* vector. The recombinant TabZIP45-4B-GST tag protein was expressed in *E.coli* BL21(DE3) strain and purified with Ni-NTA magnetic beads (genscript). The promoter of *TaDWARF4* promoter region was amplified with biotin tagged primer (Ruibio. Biotech. Beijing). The DNA shift in gel was performed according to the LightShift Chemiluminescent EMSA Kit (Thermo Fisher Scientific, Waltham, MA, USA). Related primers were listed in Supplemental Table 8.

CRISPR-Cas9-mediated gene editing

Mutant lines were created by gene editing technology (clustered regularly interspaced short palindromic repeats/associated nuclease Cas9, CRISPR/Cas9) (Shan *et al.*, 2014). Selection and design of gene target sites that are conserved in the A, B, and D genomes of each gene and specific to other homologous sequences, and the target site sequences are as follows. *TabZIP45*: TGACAGGTCCGACAGGCCTATGG. *TaDWARF4*: CCTCCTGGCCCTGCTCACCTTC.

The pTaU6-sgRNA plasmids of the respective genes were constructed separately (kindly donated by Caixia Gao). The pTaU6-sgRNA plasmids containing the target sequences and pJIT163-UBI-2NLS-rCas9-wheat (kindly donated by Caixia Gao) were commissioned to the

transformation platform of Genovo (Tianjin) to transform the wheat variety KN199WT using the gene gun transformation method as described previously (Shan *et al.*, 2014). Transgenic positive plants were obtained and firstly amplified by PCR with primers conserved in the A, B and D genomes of the respective genes. Enzyme digestion was used for identification. In case of positive mutations, the plants were then identified by PCR amplification with primers specific to the A, B and D genomes, enzymatic digestion and sequencing. Primers used were provided in Supplemental Table 8. Our optimized transgenic genome editing enables our obtaining T0 within 3-4 months.

RNA-seq and transcriptome analysis

The gene editing mutant of *TabZIP45-4B* (*Tabzip45-bb*) and wild type KN199WT were used as study materials. The root, leaf, and root-stem joint parts (shoot basal parts) samples were collected after two weeks under 0.2N and 2N culture, respectively. High-quality RNA was extracted by referring to the instructions of GeneMark's Plant Total RNA Purification Kit (TR02/TR02-150).

The library type was eukaryotic transcriptome class with fragment size between 250-300 bp. The sequencing platform was Hiseq-PE150, and the sequencing data were obtained by removing adapter sequences and low-quality sequences to obtain a data volume of > 10 Gb. The data were aligned to the reference genome database using Tophap2. All data were normalized and calculated by Deseq2 software (Love et al., 2014). Data with an error rate less than 0.05 and a difference ratio greater than log2FoldChange ≥ 1 were used to calculate the differentially expressed genes for comparison. Comparisons were made between KN199WT and the mutant *Tabzip45-bb* in the same treatment of either 0.2N or 2N culture. comparisons were made between different treatments of KN199WT or *Tabzip45-bb*.

ChIP-PCR and ChIP-Seq analysis

The procedures were done as precious described (He *et al.*, 2015; Saleh et al., 2008). Briefly, seedling under 0N condition for two weeks and whole seedlings was collected and fixed. The fixed samples were powered and lysis. The supernatant from lysis solution after centrifugated and fragmented by ultrasonic crusher was added with antibody to TabZIP45. The beads were collected after washes and then DNA was isolated. The specific primers (supplied in Table S8) were used to evaluated DNA enrichment by quantitative PCR.

The library type was eukaryotic ChIP-seq DNA library with a fragment peak size of 100 bp. Sequencing platform was Hiseq-PE150. Sequencing data were removed from splice sequences and low-quality sequences, and the sequence length was >50 bp, resulting in clean data volume >10 Gb. The database was aligned to the reference genome using BWA and enriched for target genes (Li *et al.*, 2009). The database was enriched for target genes using MACS (Deschamps et al., 2021). 63.06% were matched to the reference genome, of which 31.48% were matched to one gene and 21.35% were matched to two genes. Screening conditions: within 5 kb upstream and downstream of the gene, and there was a signal strength difference of more than 1-fold, significant q value < 0.05. primers were designed to verify target site binding ability based on ChIP-seq data. Integrative Genomics Viewer (IGV)-tools was used to visualized results (Thorvaldsdóttir et al., 2013).

Subcellular localization

The sequences of the coding regions of *TabZIP45* gene were cloned into UBI-163-GFP. The fluorescence signal was observed between 12 and 24 h after cotransformation of *TabZIP45-4B*-GFP into wheat protoplasts. The protoplasts were isolated as previously described (Fang et al., 2020a). The protoplasts were observed under a laser confocal microscope (Zeiss LSM710) for

fluorescence signals. GFP excitation light range: 500 ~ 550 nm, DAPI 350 nm, chloroplast autofluorescence excitation light range: 488 nm. Primers used were provided in Supplemental Table 8.

SNPs in different haplotypes determination assay

The different haplotypes of TabZIP45-4B seedlings were under 0N (0.0 mM NH_4NO_3) and 2N (1.0 mM NH_4NO_3) conditions for two weeks. The isolated DNA and RNA from these whole seedlings were used to determined SNPs. Related primers were listed in Supplemental Table 8.

Transcription activation assay

Luciferase activity assay in tobacco leaves was carried out according to previous methods(Han et al., 2019; Tian et al., 2024; Zhou et al., 2024). Briefly, promoter of *DWARF4* (2,500 bp) was inserted in to plasmid of pGreenII 0800-LUC as a signal reporter (Hellens et al., 2005). Coding region of bZIP45-4B haplotypes (XY54 and J411) was inserted into plasmid of pCAMBIA1300 to generated *p35S::bZIP45-4B-GFP* as an effector plasmid. These plasmids and empty vectors controls and or combinations were transformed into *A. tumefaciens* GV3101 strains. The bacteria were infiltrated into lower side of *N. benthamiana* leaves. The leaves were collected for luciferase detection according to manufactory manual of the Dual-Luciferase Reporter Assay System (Promega, E1960). At least six biological repeats from different plants were carried out to collected data, which was normalized to control of empty vectors of pGreenII 0800-LUC to calculated LUC/REN. Primers used were provided in Supplemental Table 8.

Western blotting assay

Pull off the comb from 10% NuPAGE Bis-Tris gel (Thermo Scientific™, USA), put the gel into the electrophoresis instrument, and add protein electrophoresis buffer to the electrophoresis tank. Set the voltage at 110 V. Stop electrophoresis once the target protein is expected to have been properly separated according to the protein molecular weight, and remove the gel. The membranes were placed sequentially in the pre-chilled transfer solution as blackboard-sponge-filter paper-gel-PVDF membrane-filter paper-sponge-white board (membranes were soaked in methanol for 5 min before use), and the voltage was set at 200 mA, and the membranes were transferred in an icy bath for 90 min. The PVDF membranes were removed and washed five times with TBST (50 mM Tris-HCl (pH 7.5), 8 g/L NaCl, 0.2 g/L KCl, 0.5% Tween-20) for 10 min each. Pour off TBST, add primary antibody in the appropriate proportion in TBST containing 0.5% skimmed milk powder and incubate slowly overnight at 4°C or for 2 h at 37°C. Wash the membrane 3 ~ 5 times with TBST for 10 min each time. Pour off the TBST, add secondary antibody in 0.5% skimmed milk powder in TBST in appropriate proportions and incubate for 1.5 h at 37°C with slow shaking. The membranes were washed 3 ~ 5 times with TBST for 10 min each time. The membranes were laid flat, ECL reagent was added dropwise to the membranes and photographed with Image Quant LAS 4000.

Quantitative real-time PCR (qRT-PCR)

Total RNAs were isolated from various tissues according to TRIzol™ Plus RNA Purification Kit (Invitrogen™, USA). The first strand cDNA was synthesized according to cDNA RevertAid First Strand cDNA Synthesis Kit (Thermo Scientific™, USA). The obtained cDNA samples were diluted 20-fold and quantitative PCR experiments were performed according to the instructions of the LightCycler® 480 SYBR Green I Master (Roche Molecular Systems, Inc., CHE). The specificity of the qPCR reaction was confirmed by the melting curve of the qPCR with a single peak at the end of the reaction. Calculate the mean Ct value. Determine the relative expression of the target genes based on the expression of the corresponding internal reference gene *Taactin* (*TraesCS1A02G274400*). Primers used were provided in Supplemental Table 8.

Phylogenetic analysis

The homologous protein sequences of TabZIP45 were downloaded from the database (http://plants.ensembl.org and https://www.rcsb.org). Evolutionary distances were calculated and evolutionary trees were constructed using MEGA 6.0 software for neighbor-joining (Tamura et al., 2013). Bootstrap values were 1000 times repeated. The accession numbers and databases of sequences for constructing these phylogenetic trees can be found in The Arabidopsis Information Resource database (https://www.arabidopsis.org/) and the National Center for Biotechnology Information database (https://www.ncbi.nlm.nih.gov/).

Statistical analysis and reproducibility

Numbers (n) of samples or replicates are indicated in figure legends and method section. The error bar of each bar plot indicates the s.e. in bar charts. Box plots denote the 10th percentile, the median and the 90th percentile, with minimum to maximum whiskers. For pairwise comparisons, significance analysis was calculated by two-tailed Student's *t* test using Excel 2016 and/or GraphPad Prism 8(Swift, 1997), and the exact *P* values are displayed. For multiple-group comparisons, significance analysis was calculated by one/two-way ANOVA as indicated in figure legends and indicated with different letters. All data were counted, analyzed and plotted using R (version 4.3.1) packages and/or GraphPad Prism 8 (Okoye and Hosseini, 2024; Swift, 1997). *P*-values less than 0.05 were considered to be significantly different. Appropriate statistics were selected according to the significance of the orthogonal distribution of the data and the conditions of processing. Statistical analyses for differential gene expression and bZIP45 ChIP-seq peaks calling were conducted with Deseq2 (version 3.19) R package and macs2 (macs2 2.2.4) (Feng et al., 2012) using three replicates. For SNP calling in segregating Near Isogenic Lines, we use three population to validated the genetic sites with the region around *TabZIP45-4B*.

Data and code availability

Constructs, strains, NILs, and transgenic seeds generated in this study will be made available upon request for scientific research, but a completed Materials Transfer Agreement should be required if there is potential for commercial application. All datasets have been deposited to public databases. Further information and requests for resources and reagents should be directed to and will be fulfilled by the lead contact, Yiping Tong (yptong@genetics.ac.cn). The bulk sequencing data of ChIP-seq and RNA-seq have been deposited in the Genome Sequence Archive (GSA; https://ngdc.cncb.ac.cn/gsa/) at the Beijing Institute of Genomics Data Center, Chinese Academy of Sciences, under accession number PRJCA026635 and PRJCA026636. Sequence data from this study can be found in the EnsemblPlants database (http://plants.ensembl.org) or the UniProt (https://www.uniprot.org/uniprotkb/) under the following accession number: TabZIP45-4A (TraesCS4A02G126300), TabZIP45-4B (TraesCS4B02G178600), TabZIP45-4D (TraesCS4D02G180200), TaDWARF-4A (TraesCS4A02G078000), TaDWARF-4B (TraesCS4B02G234100), TaDWARF-4D (TraesCS4D02G235200), TaActin (TraesCS1A02G274400). Sequence for constructing the phylogenetic tree of bZIPs can be found in the Arabidopsis Information Resource database (https://www.arabidopsis.org/) or the MSU database (http://rice.plantbiology.msu.edu/) or the National Center for Biotechnology Information database (https://www.ncbi.nlm.nih.gov/) or the MaizeGDB database (https://maizegdb.org/) or the EnsemblPlants. Source data are provided with this paper. Any additional information required to reanalyze the data reported in this work paper is available from the lead contact upon request.

References

An, D., Su, J., Liu, Q., Zhu, Y., Tong, Y., Li, J., Jing, R., Li, B., and Li, Z. (2006). Mapping QTLs for nitrogen uptake in relation to the early growth of wheat (Triticum aestivum L.). Plant Soil, **284**(1), 73-84.

Bolger, A.M., Lohse, M., and Usadel, B. (2014). Trimmomatic: a flexible trimmer for Illumina sequence data. Bioinformatics **30**:2114-2120.

Clevenger, J.P., and Ozias-Akins, P. (2015). SWEEP: A Tool for filtering high-quality SNPs in polyploid crops. G3 (Bethesda, Md.) **5**:1797-1803.

Deschamps, S., Crow, J.A., Chaidir, N., Peterson-Burch, B., Kumar, S., Lin, H., Zastrow-Hayes, G., and May, G.D. (2021). Chromatin loop anchors contain core structural components of the gene expression machinery in maize. BMC Genom. **22**:1-12.

Drzazga, A., Sowinska, A., Krzeminska, A., Rytczak, P., Koziolkiewicz, M., and Gendaszewska-Darmach, E. (2017). Lysophosphatidylcholine elicits intracellular calcium signaling in a GPR55-dependent manner. Biochem. Biophys. Res. Commun. **489**:242-247.

Fang, J., Zhu, W., and Tong, Y. (2020a). Knock-down the expression of brassinosteroid receptor TaBRI1 reduces photosynthesis, tolerance to high light and high temperature stresses and grain yield in wheat. Plants **9**:840.

Feng, J., Liu, T., Qin, B., Zhang, Y., and Liu, X.S. (2012). Identifying ChIP-seq enrichment using MACS. Nat. Protoc. **7**:1728-1740.

Han, X., Altegoer, F., Steinchen, W., Binnebesel, L., Schuhmacher, J., Glatter, T., Giammarinaro, P.I., Djamei, A., Rensing, S.A., and Reissmann, S. (2019). A kiwellin disarms the metabolic activity of a secreted fungal virulence factor. Nature **565**:650-653.

He, X., Qu, B., Li, W., Zhao, X., Teng, W., Ma, W., Ren, Y., Li, B., Li, Z., and Tong, Y. (2015). The nitrate-inducible NAC transcription factor TaNAC2-5A controls nitrate response and increases wheat yield. Plant physiol. **169**:1991-2005.

Hellens, R.P., Allan, A.C., Friel, E.N., Bolitho, K., Grafton, K., Templeton, M.D., Karunairetnam, S., Gleave, A.P., and Laing, W.A. (2005). Transient expression vectors for functional genomics, quantification of promoter activity and RNA silencing in plants. Plant methods **1**:1-14.

Huang, Y., Wang, H., Zhu, Y., Huang, X., Li, S., Wu, X., Zhao, Y., Bao, Z., Qin, L., and Jin, Y. (2022). THP9 enhances seed protein content and nitrogen-use efficiency in maize. Nature **612**:292-300.

Johnson, V.J., Yucesoy, B., and Luster, M.I. (2004). Genotyping of single nucleotide polymorphisms in cytokine genes using real-time PCR allelic discrimination technology. Cytokine **27**:135-141.

Kalkavan, H., Chen, M.J., Crawford, J.C., Quarato, G., Fitzgerald, P., Tait, S.W., Goding, C.R., and Green, D.R. (2022). Sublethal cytochrome c release generates drug-tolerant persister cells. Cell **185**:3356-3374.

Kim, D., Pertea, G., Trapnell, C., Pimentel, H., Kelley, R., and Salzberg, S.L. (2013). TopHat2: accurate alignment of transcriptomes in the presence of insertions, deletions and gene fusions. Genome Biol. **14**:R36.

Li, H. (2011). A statistical framework for SNP calling, mutation discovery, association mapping and population genetical parameter estimation from sequencing data. Bioinformatics **27**:2987-2993.

Li, H., Handsaker, B., Wysoker, A., Fennell, T., Ruan, J., Homer, N., Marth, G., Abecasis, G., and Durbin, R. (2009). The Sequence Alignment/Map format and SAMtools. Bioinformatics **25**:2078-2079.

Li, W., He, X., Chen, Y., Jing, Y., Shen, C., Yang, J., Teng, W., Zhao, X., Hu, W., and Hu, M. (2020). A wheat transcription factor positively sets seed vigour by regulating the grain nitrate signal. New Phytol. **225**:1667-1680.

Love, M., Anders, S., and Huber, W. (2014). Differential analysis of count data–the DESeq2 package. Genome Biol. **15**:10-1186.

Ma, F., Xu, Y., Wang, R., Tong, Y., Zhang, A., Liu, D., and An, D. (2023). Identification of major QTLs for yield-related traits with improved genetic map in wheat. Front. Plant Sci. **14**:1138696.

Martino, A., Mancuso, T., and Rossi, A.M. (2010). Application of high-resolution melting to large-scale, high-throughput SNP genotyping: a comparison with the TaqMan® method. J. Biomol. Screen. **15**:623-629.

Okoye, K., and Hosseini, S. (2024). R Programming: Statistical Data Analysis in Research

(Springer Nature).
Saleh, A., Alvarez-Venegas, R., and Avramova, Z. (2008). An efficient chromatin immunoprecipitation (ChIP) protocol for studying histone modifications in Arabidopsis plants. Nat. Protoc. **3**:1018-1025.
Shan, Q., Wang, Y., Li, J., and Gao, C. (2014). Genome editing in rice and wheat using the CRISPR/Cas system. Nat. Protoc. **9**:2395-2410.
Song, L., Liu, J., Cao, B., Liu, B., Zhang, X., Chen, Z., Dong, C., Liu, X., Zhang, Z., and Wang, W. (2023). Reducing brassinosteroid signalling enhances grain yield in semi-dwarf wheat. Nature:1-7.
Soyk, S., Lemmon, Z.H., Oved, M., Fisher, J., Liberatore, K.L., Park, S.J., Goren, A., Jiang, K., Ramos, A., and van der Knaap, E. (2017). Bypassing negative epistasis on yield in tomato imposed by a domestication gene. Cell **169**:1142-1155. e1112.
Swift, M.L. (1997). GraphPad prism, data analysis, and scientific graphing. Chem. Inf. Comput. Sci **37**:411-412.
Taira, C., Matsuda, K., Kamijyo, Y., Sakashita, K., Ishida, F., Kumagai, T., Yamauchi, K., Okumura, N., and Honda, T. (2011). Quantitative monitoring of single nucleotide mutations by allele-specific quantitative PCR can be used for the assessment of minimal residual disease in patients with hematological malignancies throughout their clinical course. Clin. Chim. Acta **412**:53-58.
Tamura, K., Stecher, G., Peterson, D., Filipski, A., and Kumar, S. (2013). MEGA6: molecular evolutionary genetics analysis version 6.0. Mol. Biol. Evol. **30**:2725-2729.
Thorvaldsdóttir, H., Robinson, J.T., and Mesirov, J.P. (2013). Integrative Genomics Viewer (IGV): high-performance genomics data visualization and exploration. Brief Bioinform. **14**:178-192.
Tian, J., Wang, C., Chen, F., Qin, W., Yang, H., Zhao, S., Xia, J., Du, X., Zhu, Y., and Wu, L. (2024). Maize smart-canopy architecture enhances yield at high densities. Nature **632**:576-584.
Xu, Y., Wang, R., Tong, Y., Zhao, H., Xie, Q., Liu, D., Zhang, A., Li, B., Xu, H., and An, D. (2014). Mapping QTLs for yield and nitrogen-related traits in wheat: influence of nitrogen and phosphorus fertilization on QTL expression. Theor. Appl. Genet. **127**:59-72.
Zhang, J., Yang, J., Zhang, L., Luo, J., Zhao, H., Zhang, J., and Wen, C. (2020). A new SNP genotyping technology Target SNP-seq and its application in genetic analysis of cucumber varieties. Sci. Rep. **10**:5623.
Zhang, N., Fan, X., Cui, F., Zhao, C., Zhang, W., Zhao, X., Yang, L., Pan, R., Chen, M., and Han, J., et al. (2017). Characterization of the temporal and spatial expression of wheat (Triticum aestivum L.) plant height at the QTL level and their influence on yield-related traits. Theor. Appl. Genet. **130**:1235-1252.
Zhao, F., Tian, S., Wu, Q., Li, Z., Ye, L., Zhuang, Y., Wang, M., Xie, Y., Zou, S., and Teng, W. (2022). Utility of Triti-Map for bulk-segregated mapping of causal genes and regulatory elements in Triticeae. Plant Commun. **3**(4):100304.
Zhou, L., Chang, G., Shen, C., Teng, W., He, X., Zhao, X., Jing, Y., Huang, Z., and Tong, Y. (2024). Functional divergences of natural variations of TaNAM-A1 in controlling leaf senescence during wheat grain filling. J. Integr. Plant Biol. **66**:1242-1260.

Table S1. Ultra high throughput and solution gene alelle discrimination technology (primers and probes) used in this study.

Table S2. Genome editing off targets verification

Table S3. P450 enzymes genes enriched by ChIP-seq

Table S4. Different expressed genes (DEGs) by TabZIP45-4B knock out under nitrogen limitation (0.2N)

Table S5. Overlap genes (between ChIP and DEGs) annotation

Table S6. Gene set enrichment analysis of gene ontology annotation.

Table S7. The perfomance of ultra-high throughput and solution gene allele discrimination technology

Table S8. Primers used in this study
