# Supplementary material for: A rare allele of TabZIP45-4B enhances wheat adaptation to low nitrogen growth conditions
Source: Plant Commun. 2026 Feb 26;7(6):101784. doi: 10.1016/j.xplc.2026.101784 (PMC13261655; doi:10.1016/j.xplc.2026.101784)
Supplement: Document S2. File S1 [file mmc2.pdf]

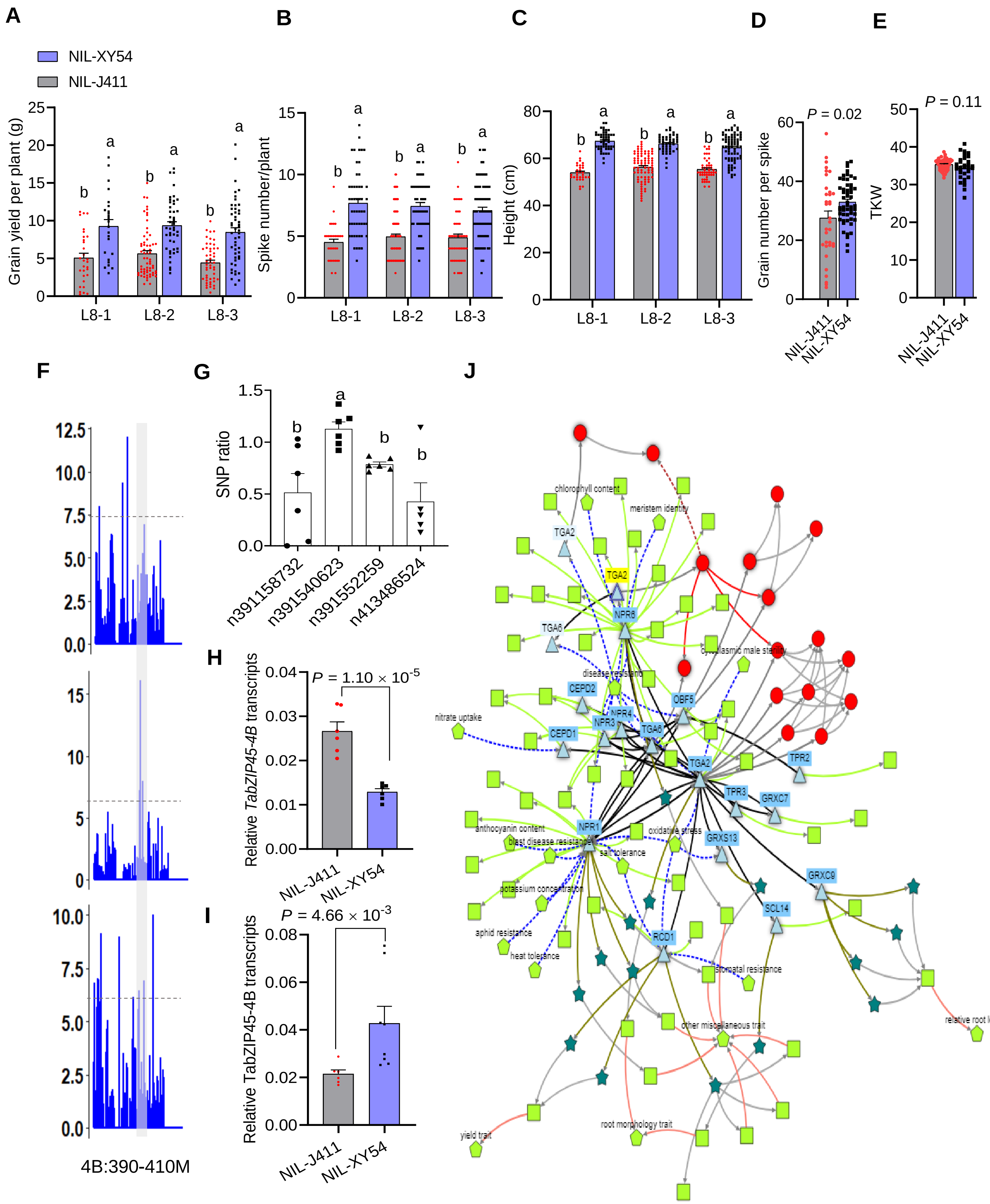

A
NIL-XY54
NIL-J411
Grain yield per plant (g)
L8-1
L8-2
L8-3
B
Spike number/plant
C
Height (cm)
D
P = 0.02
Grain number per spike
NIL-J411
NIL-XY54
E
P = 0.11
TKW
F
4B:390-410M
G
SNP ratio
n391158732
n391540623
n391552259
n413486524
H
P = 1.10 × 10-5
Relative TabZIP45-4B transcripts
I
P = 4.66 × 10-3
Relative TabZIP45-4B transcripts
J
chlorophyll content
meristem identity
TGA2
NPR6
TGA6
cytoplasmic male sterility
disease resistance
CEPD2
OBF5
NPR4
NPR3
CEPD1
nitrate uptake
TPR2
TPR3
GRXC7
NPR1
anthocyanin content
oxidative stress
blast disease resistance
salt tolerance
GRXS13
potassium concentration
GRXC9
SCL14
aphid resistance
RCD1
heat tolerance
stomatal resistance
other miscellaneous trait
relative root l
yield trait
root morphology trait

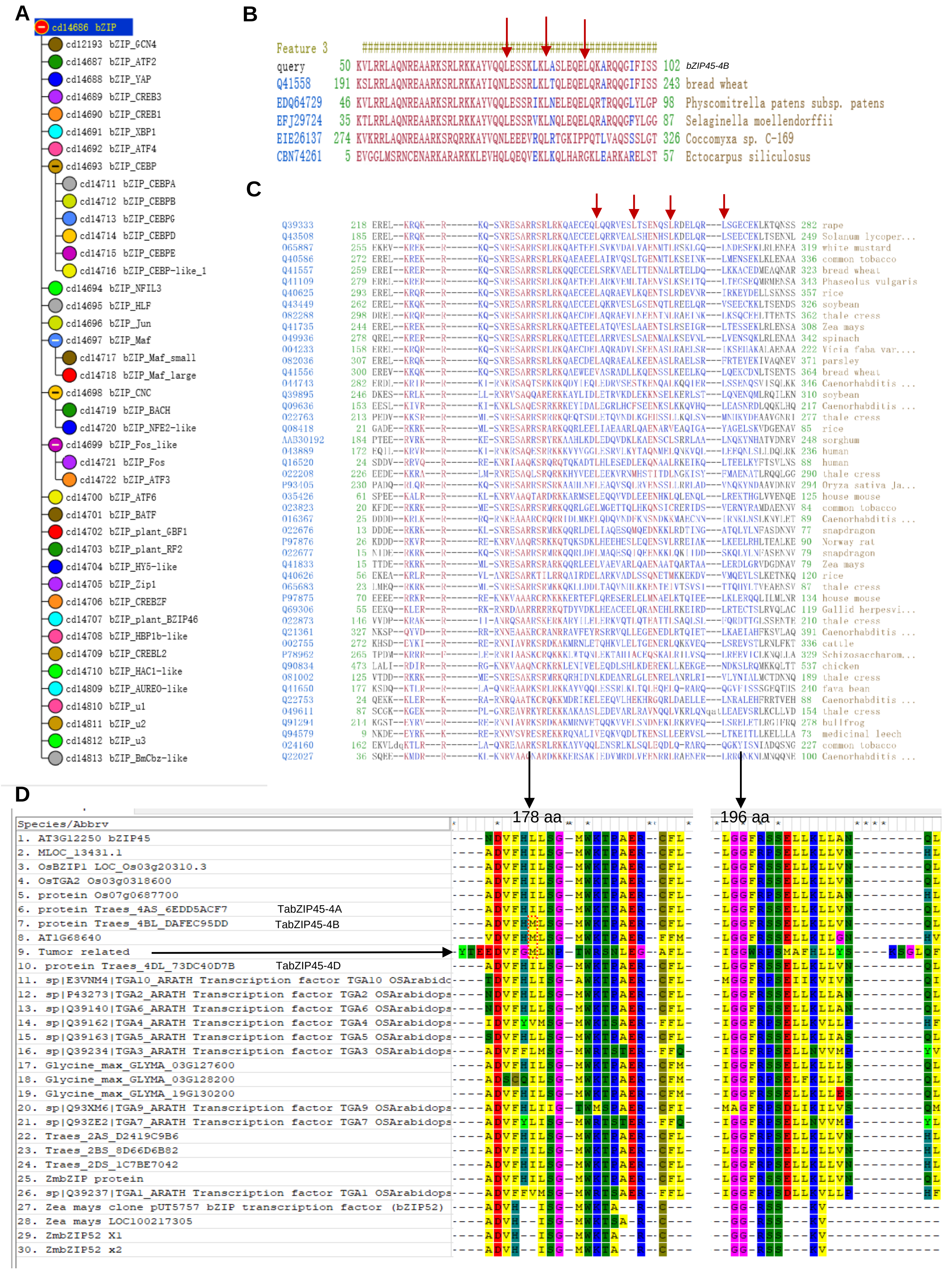
A
cd14686 bZIP
cd12193 bZIP_GCN4
cd14687 bZIP_ATF2
cd14688 bZIP_YAP
cd14689 bZIP_CREB3
cd14690 bZIP_CREB1
cd14691 bZIP_XBP1
cd14692 bZIP_ATF4
cd14693 bZIP_CEBP
cd14711 bZIP_CEBPA
cd14712 bZIP_CEBPB
cd14713 bZIP_CEBPG
cd14714 bZIP_CEBPD
cd14715 bZIP_CEBPE
cd14716 bZIP_CEBP-like_1
cd14694 bZIP_NFIL3
cd14695 bZIP_HLF
cd14696 bZIP_Jun
cd14697 bZIP_Maf
cd14717 bZIP_Maf_small
cd14718 bZIP_Maf_large
cd14698 bZIP_CNC
cd14719 bZIP_BACH
cd14720 bZIP_NFE2-like
cd14699 bZIP_Fos_like
cd14721 bZIP_Fos
cd14722 bZIP_ATF3
cd14700 bZIP_ATF6
cd14701 bZIP_BATF
cd14702 bZIP_plant_GBF1
cd14703 bZIP_plant_RF2
cd14704 bZIP_HY5-like
cd14705 bZIP_Zip1
cd14706 bZIP_CREBZF
cd14707 bZIP_plant_BZIP46
cd14708 bZIP_HBP1b-like
cd14709 bZIP_CREBL2
cd14710 bZIP_HAC1-like
cd14809 bZIP_AUREO-like
cd14810 bZIP_u1
cd14811 bZIP_u2
cd14812 bZIP_u3
cd14813 bZIP_BmCbz-like
B
Feature 3
query 50 KVLRRLAQNREAARKSRLRKKAYVQQLESSKLKLASLEQELQKARQQGIFISS 102 bZIP45-4B
Q41558 191 KSLRRLAQNREAARKSRLRKKAYIQNLESSRLKLTQLEQELQRARQQGIFISS 243 bread wheat
EDQ64729 46 KVLRRLAQNREAARKSRLRKKAYVQQLESSRIKLNELEQELQRTRQQGLYLGP 98 Physcomitrella patens subsp. patens
EFJ29724 35 KTLRRLAQNREAARKSRLRKKAYVQQLESSRVKLNQLEQELQRARQQGFYLGG 87 Selaginella moellendorffii
EIE26137 274 KVKRRLAQNREAARKSRQRRKAYVQNLEEEVRQLRTGKIPPQTLVAQSSSLGT 326 Coccomyxa sp. C-169
CBN74261 5 EVGGLMSRNCENARKARARKKLEVHQLQEQVEKLKQLHARGKLEARKARELST 57 Ectocarpus siliculosus
C
D
178 aa
196 aa
Species/Abbrv
1. AT3G12250 bZIP45
2. MLOC_13431.1
3. OsBZIP1 LOC_Os03g20310.3
4. OsTGA2 Os03g0318600
5. protein Os07g0687700
6. protein Traes_4AS_6EDD5ACF7
TabZIP45-4A
7. protein Traes_4BL_DAFEC95DD
TabZIP45-4B
8. AT1G68640
9. Tumor related
10. protein Traes_4DL_73DC40D7B
TabZIP45-4D
11. sp|E3VNM4|TGA10_ARATH Transcription factor TGA10 OSArabido
12. sp|P43273|TGA2_ARATH Transcription factor TGA2 OSArabidops
13. sp|Q39140|TGA6_ARATH Transcription factor TGA6 OSArabidops
14. sp|Q39162|TGA4_ARATH Transcription factor TGA4 OSArabidops
15. sp|Q39163|TGA5_ARATH Transcription factor TGA5 OSArabidops
16. sp|Q39234|TGA3_ARATH Transcription factor TGA3 OSArabidops
17. Glycine_max_GLYMA_03G127600
18. Glycine_max_GLYMA_03G128200
19. Glycine_max_GLYMA_19G130200
20. sp|Q93XM6|TGA9_ARATH Transcription factor TGA9 OSArabidops
21. sp|Q93ZE2|TGA7_ARATH Transcription factor TGA7 OSArabidops
22. Traes_2AS_D2419C9B6
23. Traes_2BS_8D66D6B82
24. Traes_2DS_1C7BE7042
25. ZmbZIP protein
26. sp|Q39237|TGA1_ARATH Transcription factor TGA1 OSArabidops
27. Zea mays clone pUT5757 bZIP transcription factor (bZIP52)
28. Zea mays LOC100217305
29. ZmbZIP52 X1
30. ZmbZIP52 x2

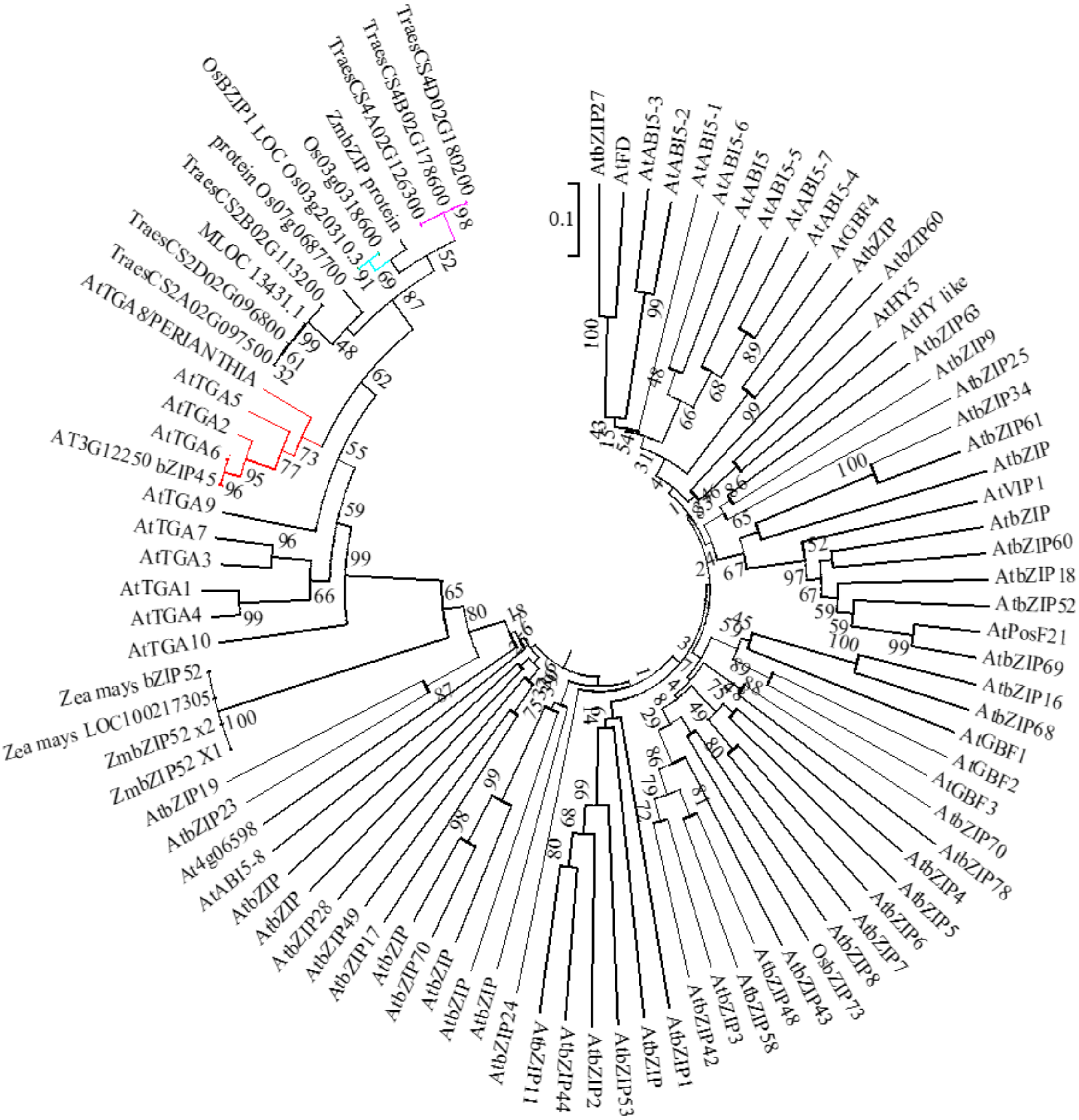


```
TabZIP45-4B    TDDKNQRMENGQNGAIVPSNSSEPSDRSDRPMDQKVLRRLAQNREAARKSRLRKKAYVQQ
               TD++ + +   GQ G   +PS+    P   +++   + +KV RR   +N+++A+ SR RKK Y+
Human CREB3    TDEEKRLL--GQEGVSLPSHL--PLTKAEERVLKKV-RRKIRNKQSAQDSRRRKKEYIDG
```

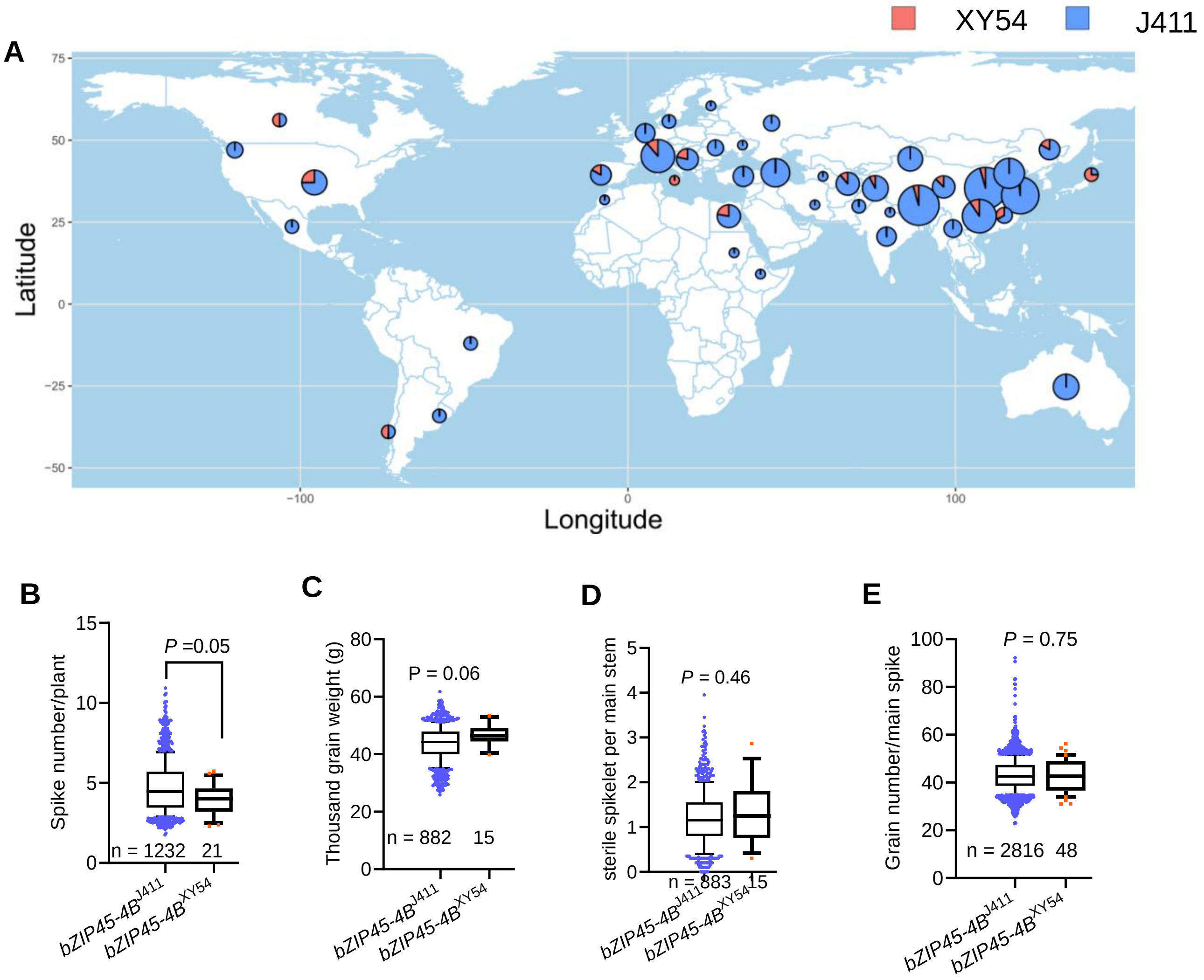
XY54
J411
A
75
50
25
0
-25
-50
Latitude
-100
0
100
Longitude
B
Spike number/plant
15
10
5
0
P =0.05
n = 1232
21
bZIP45-4B^J411
bZIP45-4B^XY54
C
Thousand grain weight (g)
80
60
40
20
0
P = 0.06
n = 882
15
bZIP45-4B^J411
bZIP45-4B^XY54
D
sterile spikelet per main stem
5
4
3
2
1
0
P = 0.46
n = 883
15
bZIP45-4B^J411
bZIP45-4B^XY54
E
Grain number/main spike
100
80
60
40
20
0
P = 0.75
n = 2816
48
bZIP45-4B^J411
bZIP45-4B^XY54

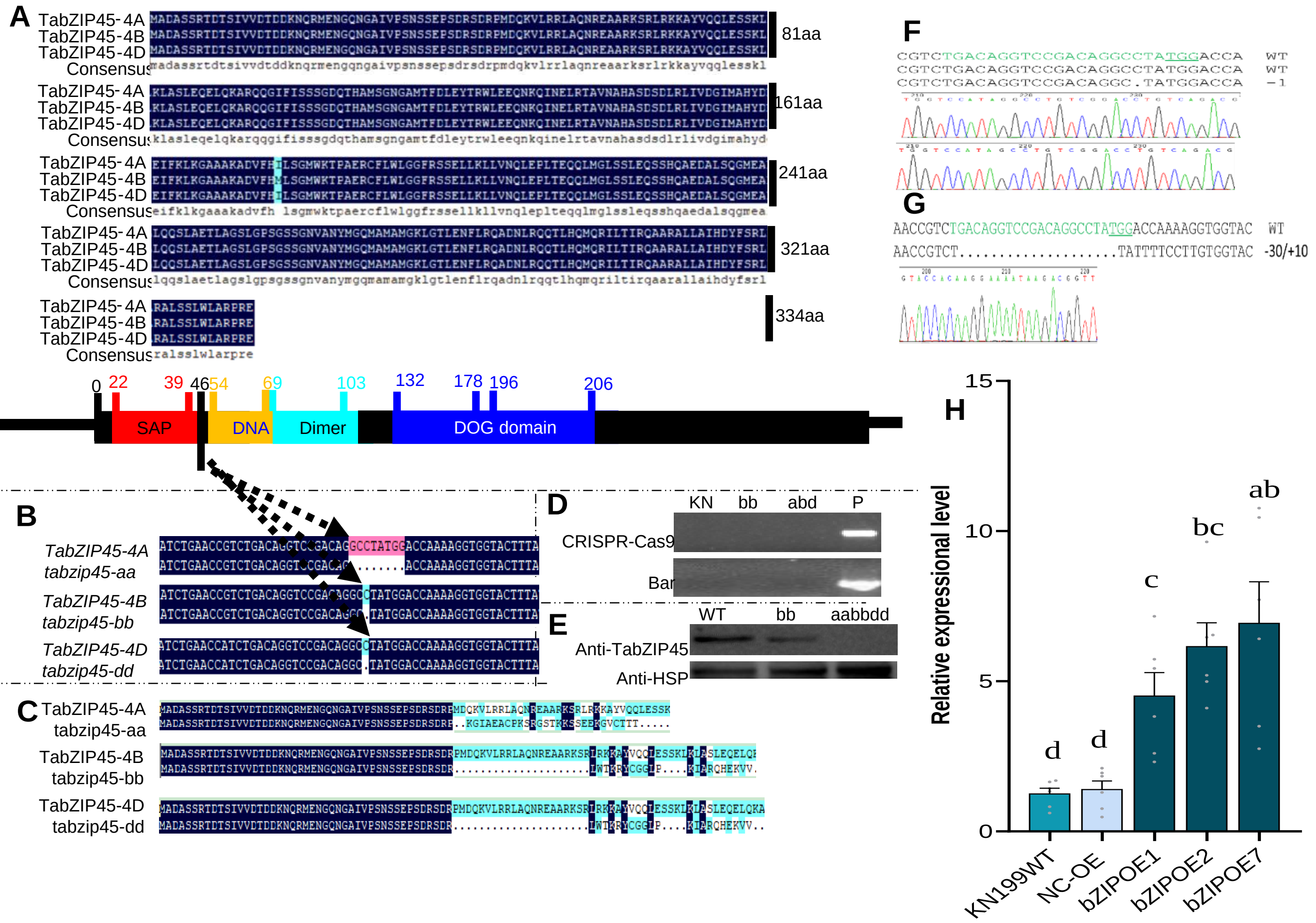
A
TabZIP45-4A
TabZIP45-4B
TabZIP45-4D
Consensus
81aa
161aa
241aa
321aa
334aa
0 22 39 46 54 69 103 132 178 196 206
SAP
DNA
Dimer
DOG domain
B
TabZIP45-4A
tabzip45-aa
TabZIP45-4B
tabzip45-bb
TabZIP45-4D
tabzip45-dd
C
TabZIP45-4A
tabzip45-aa
TabZIP45-4B
tabzip45-bb
TabZIP45-4D
tabzip45-dd
D
KN bb abd P
CRISPR-Cas9
Bar
E
WT bb aabbdd
Anti-TabZIP45
Anti-HSP
F
CGTCTGACAGGTCCGACAGGCCTATGGACCA WT
CGTCTGACAGGTCCGACAGGCCTATGGACCA WT
CGTCTGACAGGTCCGACAGGC.TATGGACCA -1
G
AACCGTCTGACAGGTCCGACAGGCCTATGGACCAAAAGGTGGTAC WT
AACCGTCT...................TATTTTCCTTGTGGTAC -30/+10
H
Relative expressional level
0
5
10
15
d
d
c
bc
ab
KN199WT
NC-OE
bZIPOE1
bZIPOE2
bZIPOE7

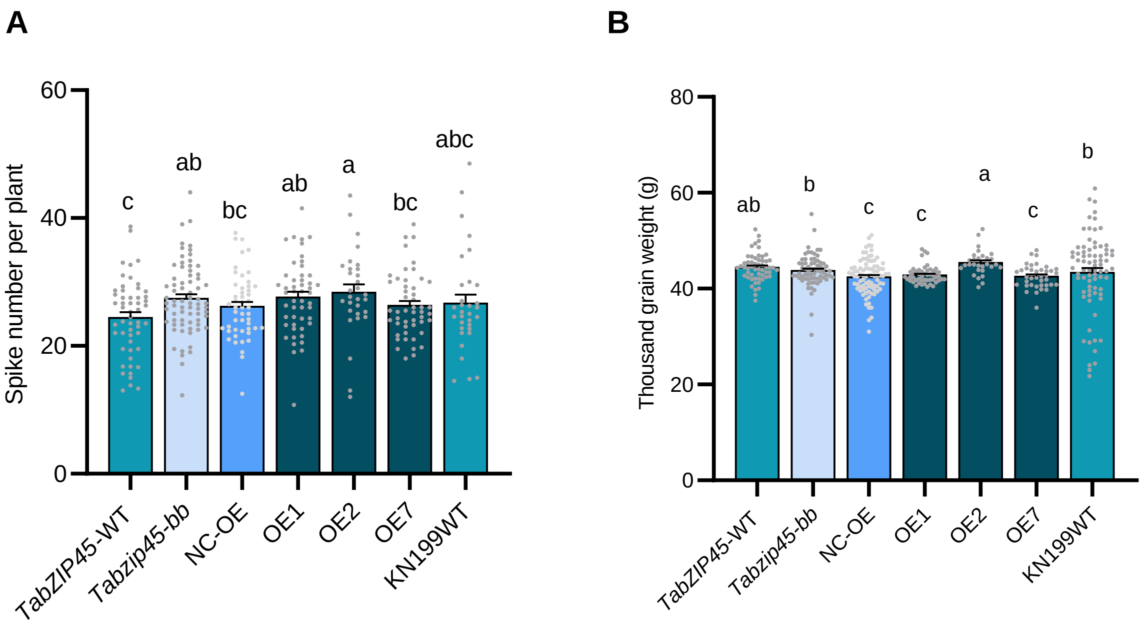
A
B
Spike number per plant
0
20
40
60
c
ab
bc
ab
a
bc
abc
TabZIP45-WT
Tabzip45-bb
NC-OE
OE1
OE2
OE7
KN199WT
Thousand grain weight (g)
0
20
40
60
80
ab
b
c
c
a
c
b
TabZIP45-WT
Tabzip45-bb
NC-OE
OE1
OE2
OE7
KN199WT

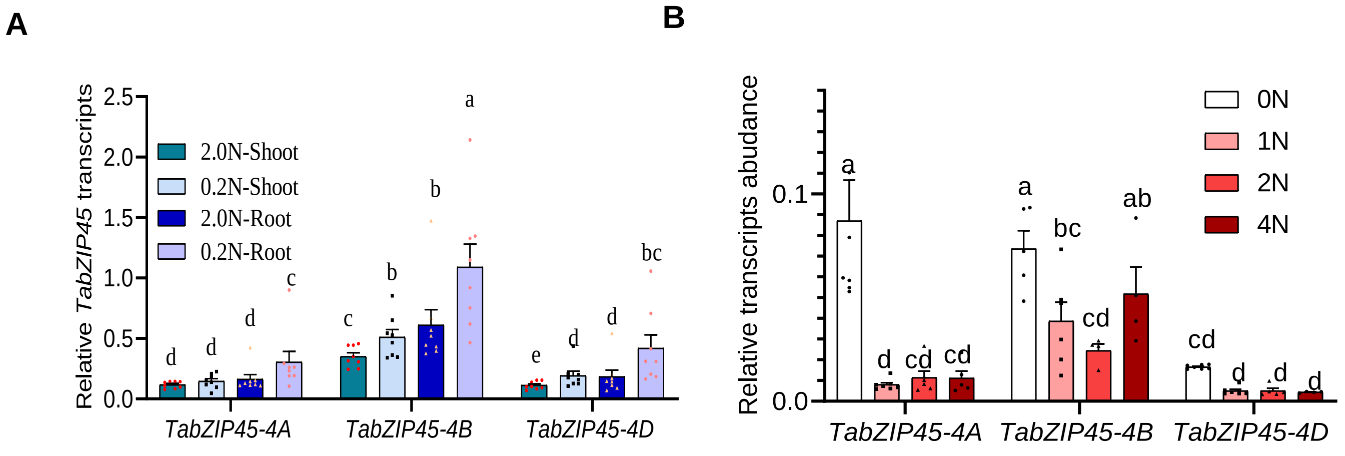

A
B
Relative TabZIP45 transcripts
2.5
2.0
1.5
1.0
0.5
0.0
2.0N-Shoot
0.2N-Shoot
2.0N-Root
0.2N-Root
TabZIP45-4A
TabZIP45-4B
TabZIP45-4D
Relative transcripts abudance
0.1
0.0
0N
1N
2N
4N
TabZIP45-4A
TabZIP45-4B
TabZIP45-4D

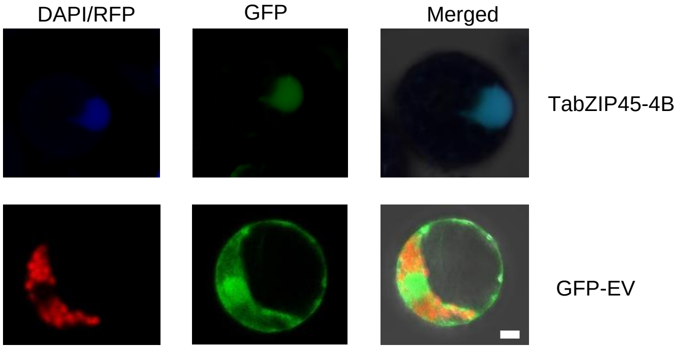
DAPI/RFP
GFP
Merged
TabZIP45-4B
GFP-EV

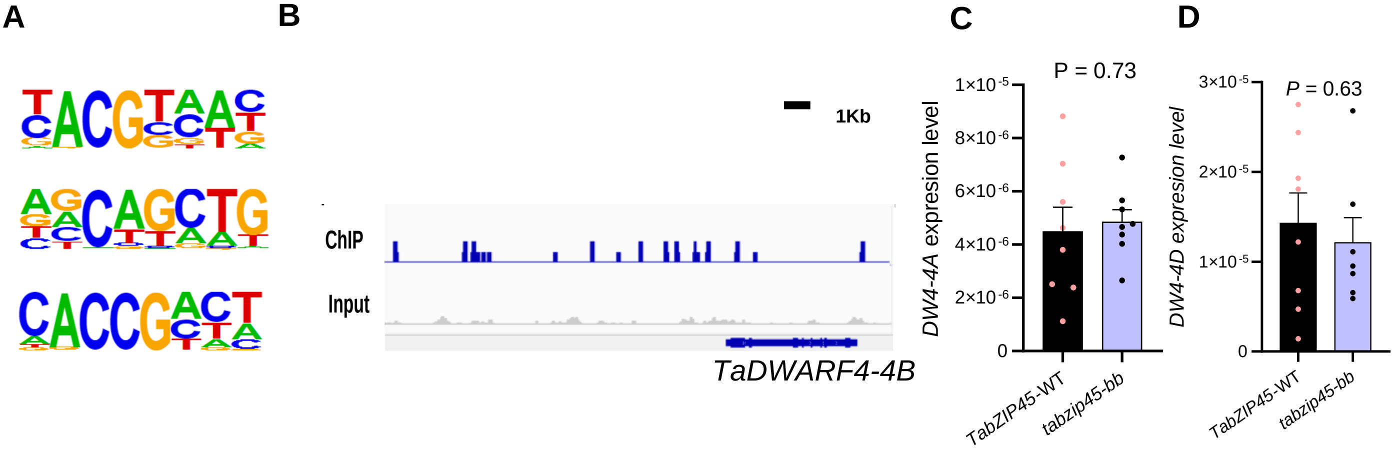
A
B
C
D
1Kb
ChIP
Input
TaDWARF4-4B
P = 0.73
DW4-4A expresion level
TabZIP45-WT
tabzip45-bb
P = 0.63
DW4-4D expresion level

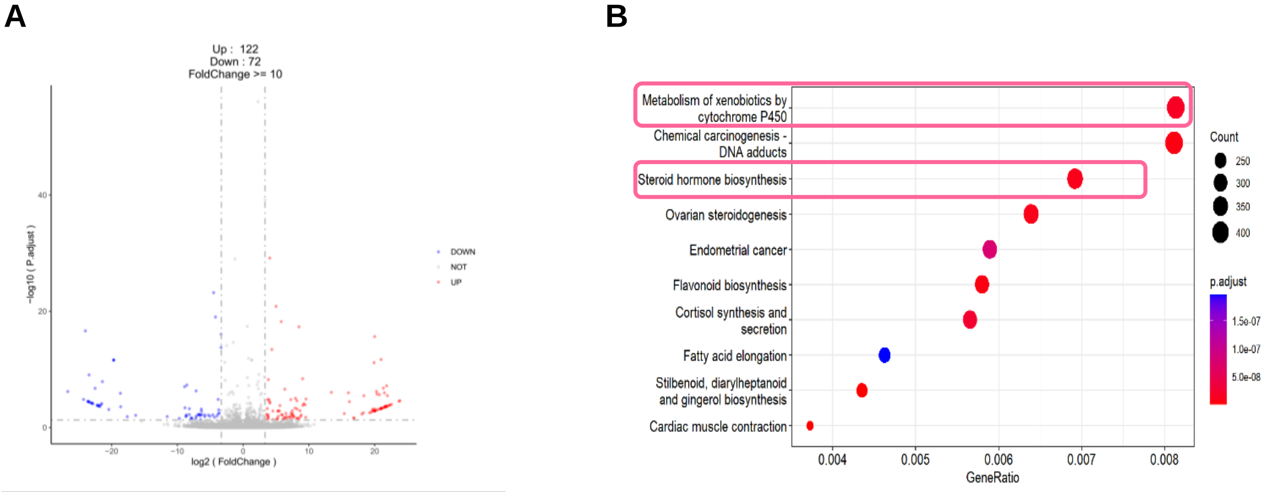
A
Up : 122
Down : 72
FoldChange >= 10
-log10 (P.adjust)
log2 (FoldChange)
DOWN
NOT
UP
B
Metabolism of xenobiotics by cytochrome P450
Chemical carcinogenesis - DNA adducts
Steroid hormone biosynthesis
Ovarian steroidogenesis
Endometrial cancer
Flavonoid biosynthesis
Cortisol synthesis and secretion
Fatty acid elongation
Stilbenoid, diarylheptanoid and gingerol biosynthesis
Cardiac muscle contraction
0.004
0.005
0.006
0.007
0.008
GeneRatio
Count
250
300
350
400
p.adjust
1.5e-07
1.0e-07
5.0e-08

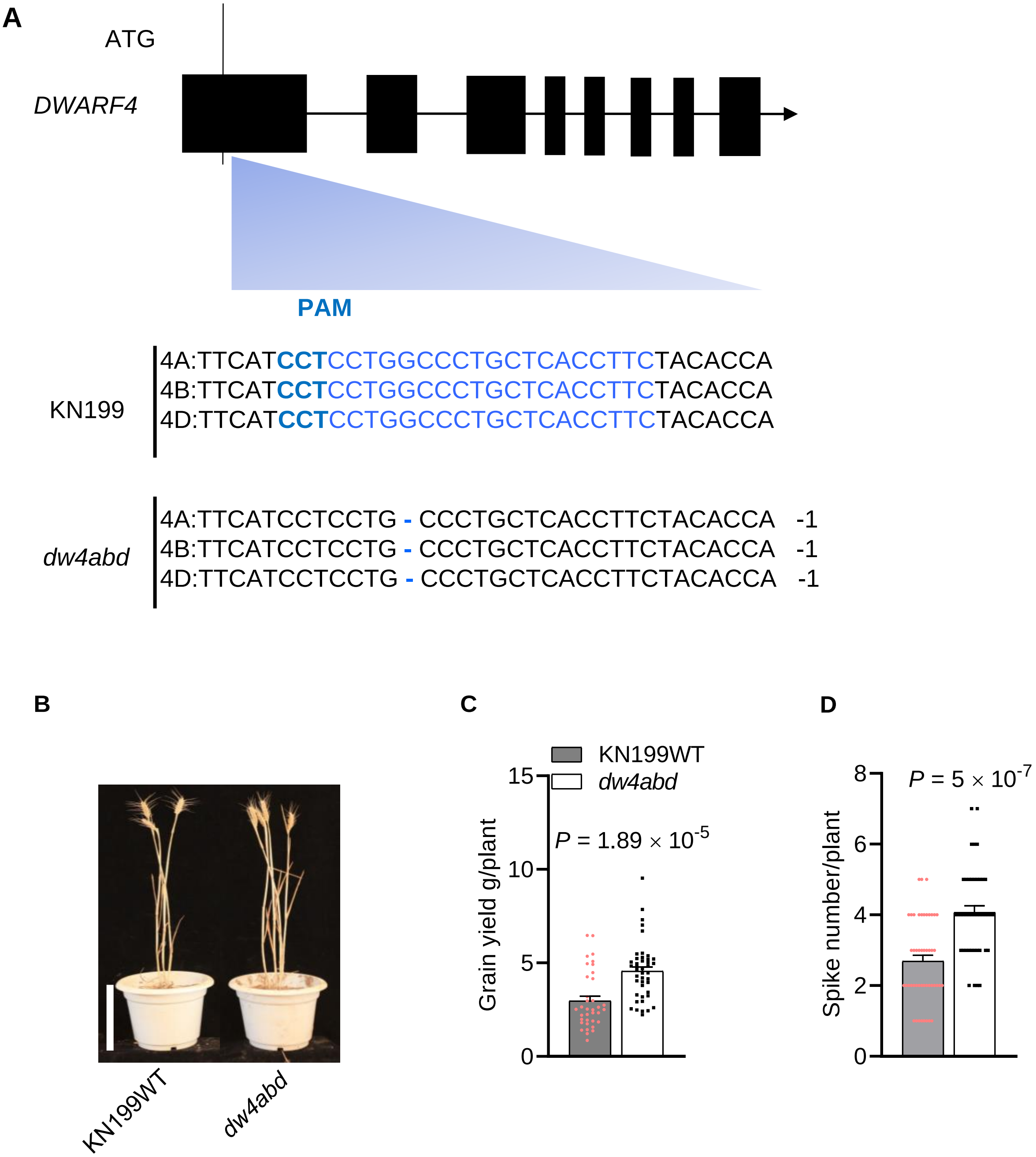
A
ATG
DWARF4
PAM
KN199
4A:TTCATCCTCCTGGCCCTGCTCACCTTCTACACCA
4B:TTCATCCTCCTGGCCCTGCTCACCTTCTACACCA
4D:TTCATCCTCCTGGCCCTGCTCACCTTCTACACCA
dw4abd
4A:TTCATCCTCCTG - CCCTGCTCACCTTCTACACCA -1
4B:TTCATCCTCCTG - CCCTGCTCACCTTCTACACCA -1
4D:TTCATCCTCCTG - CCCTGCTCACCTTCTACACCA -1
B
KN199WT
dw4abd
C
KN199WT
dw4abd
Grain yield g/plant
P = 1.89 × 10-5
0
5
10
15
D
Spike number/plant
P = 5 × 10-7
0
2
4
6
8

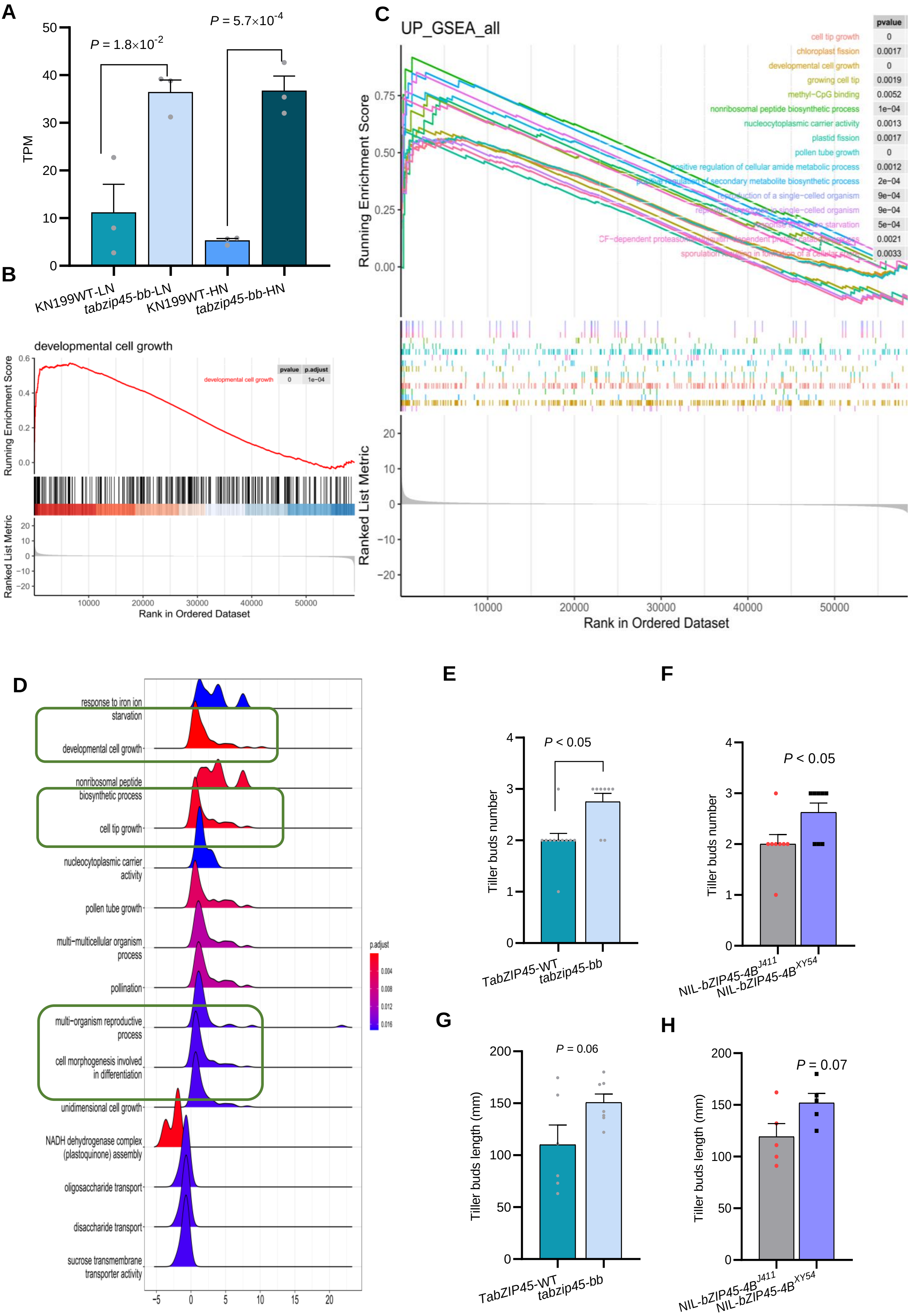
A
P = 1.8×10-2
P = 5.7×10-4
TPM
50
40
30
20
10
0
KN199WT-LN
tabzip45-bb-LN
KN199WT-HN
tabzip45-bb-HN
B
developmental cell growth
pvalue p.adjust
developmental cell growth 0 1e−04
Running Enrichment Score
Ranked List Metric
Rank in Ordered Dataset
10000
20000
30000
40000
50000
C
UP_GSEA_all
pvalue
cell tip growth 0
chloroplast fission 0.0017
developmental cell growth 0
growing cell tip 0.0019
methyl−CpG binding 0.0052
nonribosomal peptide biosynthetic process 1e−04
nucleocytoplasmic carrier activity 0.0013
plastid fission 0.0017
pollen tube growth 0
positive regulation of cellular amide metabolic process 0.0012
positive regulation of secondary metabolite biosynthetic process 2e−04
reproduction of a single−celled organism 9e−04
reproductive process in single−celled organism 9e−04
response to starvation 5e−04
SCF−dependent proteasomal ubiquitin−dependent protein catabolic process 0.0021
sporulation resulting in formation of a cellular spore 0.0033
Running Enrichment Score
Ranked List Metric
Rank in Ordered Dataset
D
response to iron ion
starvation
developmental cell growth
nonribosomal peptide biosynthetic process
cell tip growth
nucleocytoplasmic carrier activity
pollen tube growth
multi−multicellular organism process
pollination
multi−organism reproductive process
cell morphogenesis involved in differentiation
unidimensional cell growth
NADH dehydrogenase complex (plastoquinone) assembly
oligosaccharide transport
disaccharide transport
sucrose transmembrane transporter activity
p.adjust
0.004
0.008
0.012
0.016
-5
0
5
10
15
20
E
P < 0.05
Tiller buds number
TabZIP45-WT
tabzip45-bb
F
P < 0.05
Tiller buds number
NIL-bZIP45-4BJ411
NIL-bZIP45-4BXY54
G
P = 0.06
Tiller buds length (mm)
TabZIP45-WT
tabzip45-bb
H
P = 0.07
Tiller buds length (mm)
NIL-bZIP45-4BJ411
NIL-bZIP45-4BXY54

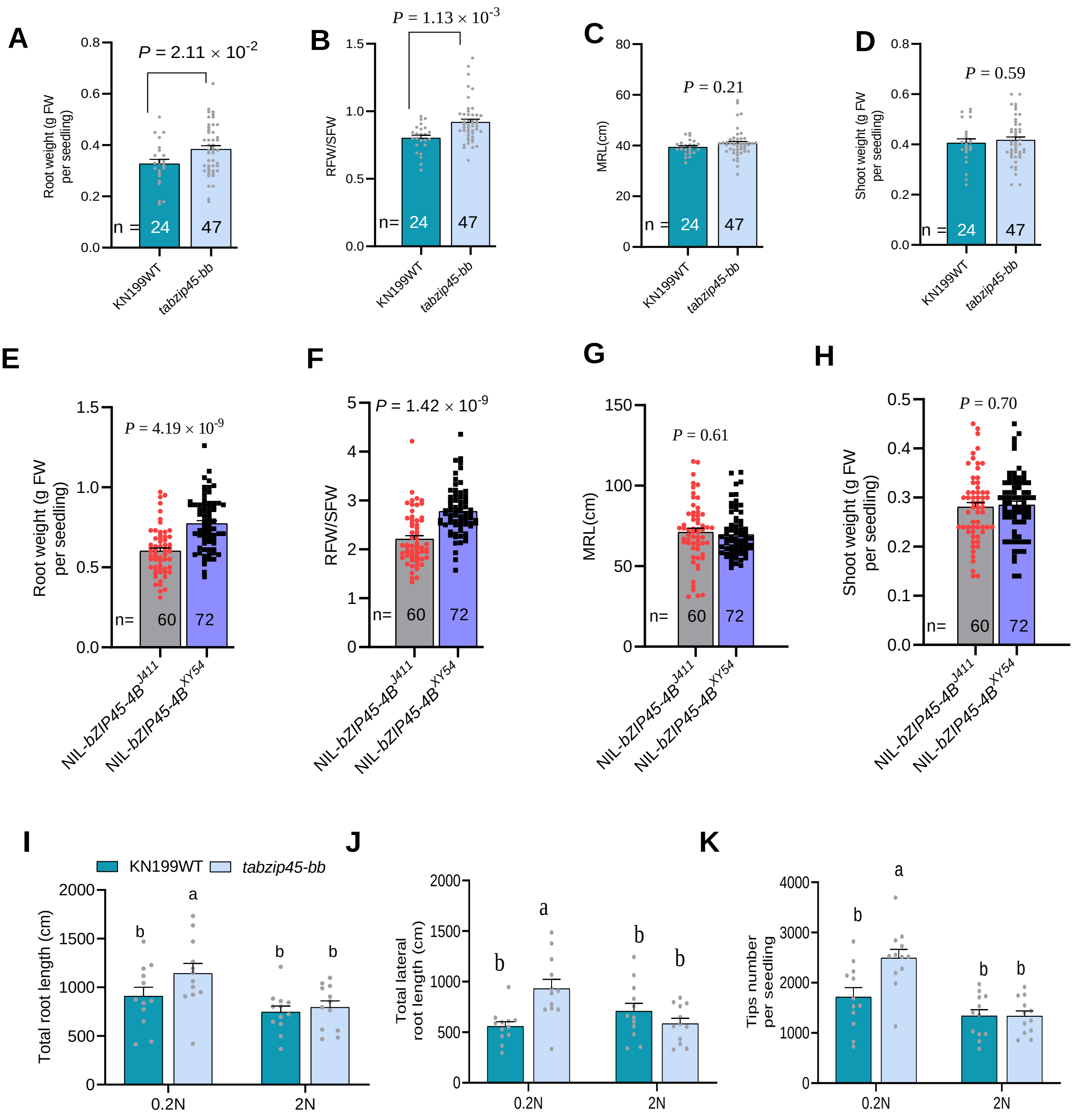
A
B
C
D
P = 2.11 × 10-2
P = 1.13 × 10-3
P = 0.21
P = 0.59
Root weight (g FW per seedling)
RFW/SFW
MRL(cm)
Shoot weight (g FW per seedling)
n = 24 47
KN199WT
tabzip45-bb
E
F
G
H
P = 4.19 × 10-9
P = 1.42 × 10-9
P = 0.61
P = 0.70
n= 60 72
NIL-bZIP45-4BJ411
NIL-bZIP45-4BXY54
I
J
K
KN199WT
tabzip45-bb
Total root length (cm)
Total lateral root length (cm)
Tips number per seedling
a
b
0.2N
2N

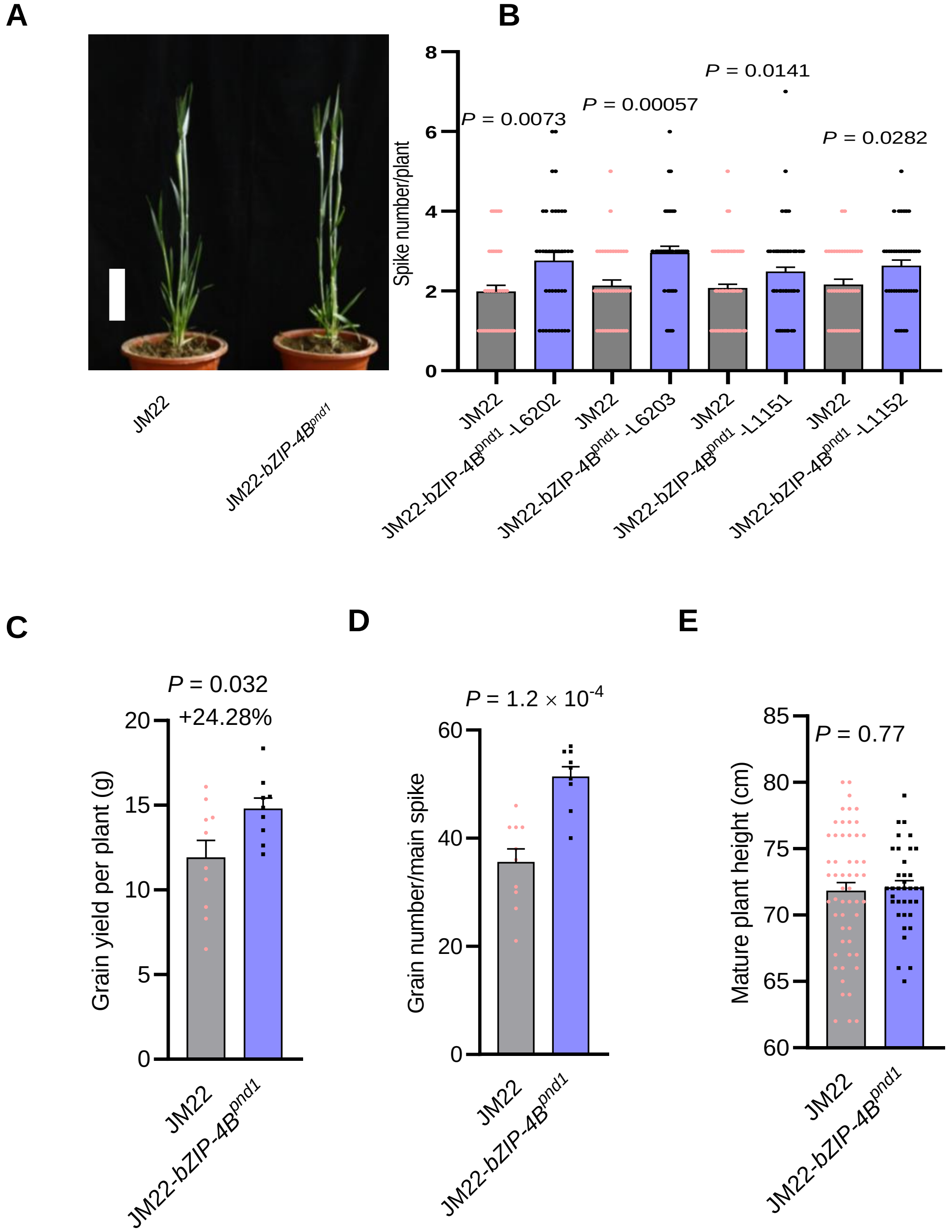
A
B
JM22
JM22-bZIP-4B^pnd1
Spike number/plant
P = 0.0073
P = 0.00057
P = 0.0141
P = 0.0282
JM22-bZIP-4B^pnd1 -L6202
JM22-bZIP-4B^pnd1 -L6203
JM22-bZIP-4B^pnd1 -L1151
JM22-bZIP-4B^pnd1 -L1152
C
D
E
P = 0.032
+24.28%
Grain yield per plant (g)
P = 1.2 × 10^-4
Grain number/main spike
P = 0.77
Mature plant height (cm)
